# Linkage disequilibrium interval mapping of quantitative trait loci

**DOI:** 10.1186/1471-2164-7-54

**Published:** 2006-03-16

**Authors:** Simon Boitard, Jihad Abdallah, Hubert de Rochambeau, Christine Cierco-Ayrolles, Brigitte Mangin

**Affiliations:** 1 Unité de Biométrie et Intelligence Artificielle, Institut National de la Recherche Agronomique, BP 52627, 31326 Castanet-Tolosan Cedex, France; 2 Laboratoire de Statistiques et Probabilités, Université Paul Sabatier, 118 route de Narbonne, 31400 Toulouse, France; 3 Laboratoire de Génétique Cellulaire, Institut National de la Recherche Agronomique, BP 52627, 31326 Castanet-Tolosan Cedex, France; 4 Station d'Amélioration Génétique des Animaux, Institut National de la Recherche Agronomique, BP 52627, 31326 Castanet-Tolosan Cedex, France

## Abstract

**Background:**

For many years gene mapping studies have been performed through linkage analyses based on pedigree data. Recently, linkage disequilibrium methods based on unrelated individuals have been advocated as powerful tools to refine estimates of gene location. Many strategies have been proposed to deal with simply inherited disease traits. However, locating quantitative trait loci is statistically more challenging and considerable research is needed to provide robust and computationally efficient methods.

**Results:**

Under a three-locus Wright-Fisher model, we derived approximate expressions for the expected haplotype frequencies in a population. We considered haplotypes comprising one trait locus and two flanking markers. Using these theoretical expressions, we built a likelihood-maximization method, called HAPim, for estimating the location of a quantitative trait locus. For each postulated position, the method only requires information from the two flanking markers. Over a wide range of simulation scenarios it was found to be more accurate than a two-marker composite likelihood method. It also performed as well as identity by descent methods, whilst being valuable in a wider range of populations.

**Conclusion:**

Our method makes efficient use of marker information, and can be valuable for fine mapping purposes. Its performance is increased if multiallelic markers are available. Several improvements can be developed to account for more complex evolution scenarios or provide robust confidence intervals for the location estimates.

## Background

The detection and mapping of loci affecting quantitative traits (QTLs) of interest in human, animal, and plant populations have attracted considerable research interest for several decades. This work has mainly concentrated on the use of pedigree or family data, especially in animal and plant populations where the structure of such experimental pedigrees can easily be planned and controlled. However, it is difficult to attain an accuracy of less than 5 centimorgans (cM) for the gene locations estimated by such linkage analysis methods because of the small number of meioses occurring in only a few generations [1,2].

More recently, linkage disequilibrium (LD) methods based on the study of unrelated individuals from a given population have emerged as a promising tool for refining gene location estimates. These methods are based on the following key hypothesis [3,4]: when a new allele is introduced into a population, either by mutation or migration, it exists in that population with a unique set of marker alleles. The length of this characteristic haplotype is then reduced along generations by recombination events, and after many generations only the markers in the immediate vicinity of the new allele locus are likely to remain on the same strand. If the new allele has a particular influence on a given trait, a strong correlation between this trait value and a marker allele might thus indicate that the coding locus is very close to the marker.

In practice, the earlier successes in mapping genes using such strategies concerned simply inherited (Mendelian) disease genes in isolated human populations [3,5-7], and the many mapping methods that have been subsequently developed for this kind of problem can be roughly divided into two classes: (i) forward analyses of allele or haplotype frequencies in the disease (case) and normal (control) populations [8-13], and (ii) backward inferences of the case sample genealogy using coalescence [14-16]. Some of these methods are specifically designed for populations divided into cases and controls, and take advantage of the assumption that the allele responsible for the disease is rare. Consequently, they are difficult to extend to mapping QTLs or complex disease traits.

The association between a quantitative trait and a marker allele can be exploited in QTL mapping. This was first proposed in [17] through a simple analysis-of-variance framework. We [18] and Farnir and colleagues [19] subsequently used a maximum-likelihood approach, based on the same kind of allele frequency model as in [9] but for the purpose of QTL mapping. Pérez-Encizo [20] provided a method based on a hidden Markov model for marker identity by descent (IBD) with the ancestral haplotype [13]. Meuwissen and Goddard [21,22] integrated the LD information in a mixed linear model through a matrix of IBD probabilities for the sample marker haplotypes. They used the so-called gene-dropping method and approximate theoretical expressions to compute these probabilities. More recently, Zöllner and Pritchard [23] developed a Bayesian method based on backward simulations of the sample ancestry using a local approximation of the ancestral recombination graph [24]. Encouraging results were also obtained in practice. For instance, an allele substitution that has a major effect on milk yield and composition was identified using LD information [25]. The present interest in finding new associations is fuelled by the increasing number of new polymorphic markers available on human and livestock genomes. However, QTL mapping remains a statistical challenge due to the weak phenotype-genotype correlation and the influence of environmental or multigene factors. Furthermore, the accuracy and computational efficiency of mapping methods still need to be increased.

Our method is an interval-mapping method designed for unrelated individuals with no family information, and is based on a maximum-likelihood calculation. Computations of the likelihood function at each postulated location of the QTL rely on the expected frequencies of a three-locus haplotype composed of the QTL and its two flanking markers. We provide an approximate expression of these expected frequencies at time *t*, assuming a Wright-Fisher model for the population and a punctual creation of LD at time 0, as described above. Due to this approximation the computation time required by our method is very low.

In this paper, we first describe the model we use and explain the differences between our method and existing ones. We then report the results of a simulation study, in which we test our method under various evolution scenarios, and compare it with the composite two-marker method in [18] and the multimarker methods in [21,26,27]. Finally we discuss the advantages and drawbacks of our method, as well as the potential improvements that could be implemented.

## Results

### Maximum likelihood approach

We consider a single quantitative trait whose value is partly controlled by a biallelic locus with alleles *Q *and *q*. As usual (and following [28]), the probability density of phenotype *Y *conditional on QTL genotype **G **is modeled as follows:

dℙ(Y=y|G)={φμ+a,σ2(y)if G=Q/Qφμ+a,σ2(y)if G=q/qφμ+d,σ2(y)if G=Q/q     (1)
 MathType@MTEF@5@5@+=feaafiart1ev1aaatCvAUfKttLearuWrP9MDH5MBPbIqV92AaeXatLxBI9gBamrtHrhAL1wy0L2yHvtyaeHbnfgDOvwBHrxAJfwnaebbnrfifHhDYfgasaacH8akY=wiFfYdH8Gipec8Eeeu0xXdbba9frFj0=OqFfea0dXdd9vqai=hGuQ8kuc9pgc9s8qqaq=dirpe0xb9q8qiLsFr0=vr0=vr0dc8meaabaqaciaacaGaaeqabaWaaeGaeaaakeaacqqGKbazt0uy0HwzTfgDPnwy3aqeh0uy0HwzTfgDPnwy3aacfaGae8xgHaLaeiikaGIaemywaKLaeyypa0JaemyEaKNaeiiFaW3exLMBbXgBcf2CPn2qVrwzqf2zLnharGGvLjhzH5wyaGGbbiaa+DeacqGGPaqkcqGH9aqpdaGabaqaauaabeqadiaaaeaaiiGacqqFgpGzdaWgaaWcbaGae0hVd0Maey4kaSIaemyyaeMaeiilaWIae03Wdm3aaWbaaWqabeaacqaIYaGmaaaaleqaaOGaeiikaGIaemyEaKNaeiykaKcabaGaeeyAaKMaeeOzayMaeeiiaaIaa43raiabg2da9iabdgfarjabc+caViabdgfarbqaaiab9z8aMnaaBaaaleaacqqF8oqBcqGHRaWkcqWGHbqycqGGSaalcqqFdpWCdaahaaadbeqaaiabikdaYaaaaSqabaGccqGGOaakcqWG5bqEcqGGPaqkaeaacqqGPbqAcqqGMbGzcqqGGaaicaGFhbGaeyypa0JaemyCaeNaei4la8IaemyCaehabaGae0NXdy2aaSbaaSqaaiab9X7aTjabgUcaRiabdsgaKjabcYcaSiab9n8aZnaaCaaameqabaGaeGOmaidaaaWcbeaakiabcIcaOiabdMha5jabcMcaPaqaaiabbMgaPjabbAgaMjabbccaGiaa+DeacqGH9aqpcqWGrbqucqGGVaWlcqWGXbqCaaaacaGL7baacaWLjaGaaCzcamaabmaabaGaeGymaedacaGLOaGaayzkaaaaaa@9AC2@

where φm,σ2
 MathType@MTEF@5@5@+=feaafiart1ev1aaatCvAUfKttLearuWrP9MDH5MBPbIqV92AaeXatLxBI9gBaebbnrfifHhDYfgasaacH8akY=wiFfYdH8Gipec8Eeeu0xXdbba9frFj0=OqFfea0dXdd9vqai=hGuQ8kuc9pgc9s8qqaq=dirpe0xb9q8qiLsFr0=vr0=vr0dc8meaabaqaciaacaGaaeqabaqabeGadaaakeaaiiGacqWFgpGzdaWgaaWcbaGaemyBa0MaeiilaWIae83Wdm3aaWbaaWqabeaacqaIYaGmaaaaleqaaaaa@33C4@ is the density function of a normal distribution N
 MathType@MTEF@5@5@+=feaafiart1ev1aaatCvAUfKttLearuWrP9MDH5MBPbIqV92AaeXatLxBI9gBamrtHrhAL1wy0L2yHvtyaeHbnfgDOvwBHrxAJfwnaebbnrfifHhDYfgasaacH8akY=wiFfYdH8Gipec8Eeeu0xXdbba9frFj0=OqFfea0dXdd9vqai=hGuQ8kuc9pgc9s8qqaq=dirpe0xb9q8qiLsFr0=vr0=vr0dc8meaabaqaciaacaGaaeqabaWaaeGaeaaakeaaimaacqWFneVtaaa@383B@(*m*, *σ*^2^) *a *is the additive effect of the QTL, *d *is the dominance effect, and *μ *is the mean trait value for homozygotes.

Our data contain *N*_*s *_unrelated individuals sampled from the same population. We observe their phenotypic values *y*_*n*_, *n *= 1,…, *N*_*s*_, and their genotypes **m**_*n *_for a given set of markers. For the purpose of generality, we do not yet specify how many of these markers there are. Our aim is to estimate as accurately as possible the position *x *of the QTL on the known marker map, for which we use a multipoint approach consisting of computing – for a large number of positions *x *of the QTL – the likelihood function ℒ
 MathType@MTEF@5@5@+=feaafiart1ev1aaatCvAUfKttLearuWrP9MDH5MBPbIqV92AaeXatLxBI9gBamrtHrhAL1wy0L2yHvtyaeHbnfgDOvwBHrxAJfwnaebbnrfifHhDYfgasaacH8akY=wiFfYdH8Gipec8Eeeu0xXdbba9frFj0=OqFfea0dXdd9vqai=hGuQ8kuc9pgc9s8qqaq=dirpe0xb9q8qiLsFr0=vr0=vr0dc8meaabaqaciaacaGaaeqabaWaaeGaeaaakeaaimaacqWFsectaaa@376E@(*x *| D
 MathType@MTEF@5@5@+=feaafiart1ev1aaatCvAUfKttLearuWrP9MDH5MBPbIqV92AaeXatLxBI9gBamrtHrhAL1wy0L2yHvtyaeHbnfgDOvwBHrxAJfwnaebbnrfifHhDYfgasaacH8akY=wiFfYdH8Gipec8Eeeu0xXdbba9frFj0=OqFfea0dXdd9vqai=hGuQ8kuc9pgc9s8qqaq=dirpe0xb9q8qiLsFr0=vr0=vr0dc8meaabaqaciaacaGaaeqabaWaaeGaeaaakeaaimaacqWFdepraaa@3827@), where D
 MathType@MTEF@5@5@+=feaafiart1ev1aaatCvAUfKttLearuWrP9MDH5MBPbIqV92AaeXatLxBI9gBamrtHrhAL1wy0L2yHvtyaeHbnfgDOvwBHrxAJfwnaebbnrfifHhDYfgasaacH8akY=wiFfYdH8Gipec8Eeeu0xXdbba9frFj0=OqFfea0dXdd9vqai=hGuQ8kuc9pgc9s8qqaq=dirpe0xb9q8qiLsFr0=vr0=vr0dc8meaabaqaciaacaGaaeqabaWaaeGaeaaakeaaimaacqWFdepraaa@3827@ = {(*y*_*n*_, **m**_*n*_), *n *= 1,…, *N*_*s*_}. The value of *x *that maximizes this likelihood function will be the estimate of the QTL position.

Since individuals are unrelated, the pairs of random variables (*Y*_*n*_, **M**_*n*_) can be considered as independent. Therefore, the likelihood function is

ℒ(x|D)=∏n=1Nsdℙ(Yn=yn,Mn=mn|x)∝∏n=1Nsdℙ(Yn=yn|Mn=mn,x)
 MathType@MTEF@5@5@+=feaafiart1ev1aaatCvAUfKttLearuWrP9MDH5MBPbIqV92AaeXatLxBI9gBamrtHrhAL1wy0L2yHvtyaeHbnfgDOvwBHrxAJfwnaebbnrfifHhDYfgasaacH8akY=wiFfYdH8Gipec8Eeeu0xXdbba9frFj0=OqFfea0dXdd9vqai=hGuQ8kuc9pgc9s8qqaq=dirpe0xb9q8qiLsFr0=vr0=vr0dc8meaabaqaciaacaGaaeqabaWaaeGaeaaakeaaimaacqWFsectcqGGOaakcqWG4baEcqGG8baFcqWFdeprcqGGPaqkcqGH9aqpdaqeWbqaaiabbsgaKnrtHrhAL1wy0L2yHDdarCqtHrhAL1wy0L2yHDdaiuaacqGFzecuaSqaaiabd6gaUjabg2da9iabigdaXaqaaiabd6eaonaaBaaameaacqWGZbWCaeqaaaqdcqGHpis1aOGaeiikaGIaemywaK1aaSbaaSqaaiabd6gaUbqabaGccqGH9aqpcqWG5bqEdaWgaaWcbaGaemOBa4gabeaakiabcYcaSmXvP5wqSXMqHnxAJn0BKvguHDwzZbqeiyvzYrwyUfgaiyqacaqFnbWaaSbaaSqaaiabd6gaUbqabaGccqGH9aqpcaqFTbWaaSbaaSqaaiabb6gaUbqabaGccqGG8baFcqWG4baEcqGGPaqkcqGHDisTdaqeWbqaaiabbsgaKjab+LriqjabcIcaOiabdMfaznaaBaaaleaacqWGUbGBaeqaaaqaaiabd6gaUjabg2da9iabigdaXaqaaiabd6eaonaaBaaameaacqWGZbWCaeqaaaqdcqGHpis1aOGaeyypa0JaemyEaK3aaSbaaSqaaiabd6gaUbqabaGccqGG8baFcaqFnbWaaSbaaSqaaiabd6gaUbqabaGccqGH9aqpcaqFTbWaaSbaaSqaaiabb6gaUbqabaGccqGGSaalcqWG4baEcqGGPaqkaaa@8E7F@

where ∝ means "proportional to", since the multiplicative constant is independent of *x*. We exploit the parametric model (1) by deriving the probabilities d(*y*_*n *_= *y*_*n*_, **M**_*n *_= **m**_*n *_| *x*), *n *= 1,…, *N*_*s*_, conditional on the random variables **G**_*n *_that denote the QTL genotype for individual *n*. We get for all *n *that

dℙ(Yn=yn|Mn=mn,x)=φμ+a,σ2(yn)ℙ(Gn=Q/Q|Mn=mn,x)+φμ−a,σ2(yn)ℙ(Gn=q/q|Mn=mn,x)+φμ+d,σ2(yn)ℙ(Gn=Q/q|Mn=mn,x)
 MathType@MTEF@5@5@+=feaafiart1ev1aaatCvAUfKttLearuWrP9MDH5MBPbIqV92AaeXatLxBI9gBamrtHrhAL1wy0L2yHvtyaeHbnfgDOvwBHrxAJfwnaebbnrfifHhDYfgasaacH8akY=wiFfYdH8Gipec8Eeeu0xXdbba9frFj0=OqFfea0dXdd9vqai=hGuQ8kuc9pgc9s8qqaq=dirpe0xb9q8qiLsFr0=vr0=vr0dc8meaabaqaciaacaGaaeqabaWaaeGaeaaakeaafaqabeWadaaabaGaeeizaq2enfgDOvwBHrxAJf2naeXbnfgDOvwBHrxAJf2naGqbaiab=LriqjabcIcaOiabdMfaznaaBaaaleaacqWGUbGBaeqaaOGaeyypa0JaemyEaK3aaSbaaSqaaiabd6gaUbqabaGccqGG8baFtCvAUfeBSjuyZL2yd9gzLbvyNv2CaebcwvMCKfMBHbacgeGaa4xtamaaBaaaleaacqWGUbGBaeqaaOGaeyypa0Jaa4xBamaaBaaaleaacqWGUbGBaeqaaOGaeiilaWIaemiEaGNaeiykaKcabaGaeyypa0dabaacciGae0NXdy2aaSbaaSqaaiab9X7aTjabgUcaRiabdggaHjab9XcaSiab9n8aZnaaCaaameqabaGaeGOmaidaaaWcbeaakiabcIcaOiabdMha5naaBaaaleaacqWGUbGBaeqaaOGaeiykaKIae8xgHaLaeiikaGIaa43ramaaBaaaleaacqWGUbGBaeqaaOGaeyypa0JaemyuaeLaei4la8IaemyuaeLaeiiFaWNaa4xtamaaBaaaleaacqWGUbGBaeqaaOGaeyypa0Jaa4xBamaaBaaaleaacqWGUbGBcqGGSaalcqWG4baEaeqaaOGaeiykaKcabaaabaGaey4kaScabaGae0NXdy2aaSbaaSqaaiab9X7aTjabgkHiTiabdggaHjab9XcaSiab9n8aZnaaCaaameqabaGaeGOmaidaaaWcbeaakiabcIcaOiabdMha5naaBaaaleaacqWGUbGBaeqaaOGaeiykaKIae8xgHaLaeiikaGIaa43ramaaBaaaleaacqWGUbGBaeqaaOGaeyypa0JaemyCaeNaei4la8IaemyCaeNaeiiFaWNaa4xtamaaBaaaleaacqWGUbGBaeqaaOGaeyypa0Jaa4xBamaaBaaaleaacqWGUbGBcqGGSaalcqWG4baEaeqaaOGaeiykaKcabaaabaGaey4kaScabaGae0NXdy2aaSbaaSqaaiab9X7aTjabgUcaRiabdsgaKjab9XcaSiab9n8aZnaaCaaameqabaGaeGOmaidaaaWcbeaakiabcIcaOiabdMha5naaBaaaleaacqWGUbGBaeqaaOGaeiykaKIae8xgHaLaeiikaGIaa43ramaaBaaaleaacqWGUbGBaeqaaOGaeyypa0JaemyuaeLaei4la8IaemyCaeNaeiiFaWNaa4xtamaaBaaaleaacqWGUbGBaeqaaOGaeyypa0Jaa4xBamaaBaaaleaacqWGUbGBcqGGSaalcqWG4baEaeqaaOGaeiykaKcaaaaa@C766@

Let us now assume that the haplotype phases are known. Each genotype **m**_*n *_can thus be written as the diplotype hn1
 MathType@MTEF@5@5@+=feaafiart1ev1aaatCvAUfKttLearuWrP9MDH5MBPbIqV92AaeXatLxBI9gBaebbnrfifHhDYfgasaacH8akY=wiFfYdH8Gipec8Eeeu0xXdbba9frFj0=OqFfea0dXdd9vqai=hGuQ8kuc9pgc9s8qqaq=dirpe0xb9q8qiLsFr0=vr0=vr0dc8meaabaqaciaacaGaaeqabaqabeGadaaakeaatCvAUfeBSjuyZL2yd9gzLbvyNv2CaeHbwvMCKfMBHbaceeGaa8hAamaaDaaaleaacqWGUbGBaeaacqaIXaqmaaaaaa@3AA3@/hn2
 MathType@MTEF@5@5@+=feaafiart1ev1aaatCvAUfKttLearuWrP9MDH5MBPbIqV92AaeXatLxBI9gBaebbnrfifHhDYfgasaacH8akY=wiFfYdH8Gipec8Eeeu0xXdbba9frFj0=OqFfea0dXdd9vqai=hGuQ8kuc9pgc9s8qqaq=dirpe0xb9q8qiLsFr0=vr0=vr0dc8meaabaqaciaacaGaaeqabaqabeGadaaakeaatCvAUfeBSjuyZL2yd9gzLbvyNv2CaeHbwvMCKfMBHbaceeGaa8hAamaaDaaaleaacqWGUbGBaeaacqaIYaGmaaaaaa@3AA5@, where hn1
 MathType@MTEF@5@5@+=feaafiart1ev1aaatCvAUfKttLearuWrP9MDH5MBPbIqV92AaeXatLxBI9gBaebbnrfifHhDYfgasaacH8akY=wiFfYdH8Gipec8Eeeu0xXdbba9frFj0=OqFfea0dXdd9vqai=hGuQ8kuc9pgc9s8qqaq=dirpe0xb9q8qiLsFr0=vr0=vr0dc8meaabaqaciaacaGaaeqabaqabeGadaaakeaatCvAUfeBSjuyZL2yd9gzLbvyNv2CaeHbwvMCKfMBHbaceeGaa8hAamaaDaaaleaacqWGUbGBaeaacqaIXaqmaaaaaa@3AA3@ and hn2
 MathType@MTEF@5@5@+=feaafiart1ev1aaatCvAUfKttLearuWrP9MDH5MBPbIqV92AaeXatLxBI9gBaebbnrfifHhDYfgasaacH8akY=wiFfYdH8Gipec8Eeeu0xXdbba9frFj0=OqFfea0dXdd9vqai=hGuQ8kuc9pgc9s8qqaq=dirpe0xb9q8qiLsFr0=vr0=vr0dc8meaabaqaciaacaGaaeqabaqabeGadaaakeaatCvAUfeBSjuyZL2yd9gzLbvyNv2CaeHbwvMCKfMBHbaceeGaa8hAamaaDaaaleaacqWGUbGBaeaacqaIYaGmaaaaaa@3AA5@ belong to the set of all haplotypes that can be found in the population for the *L *marker loci. Let jn1
 MathType@MTEF@5@5@+=feaafiart1ev1aaatCvAUfKttLearuWrP9MDH5MBPbIqV92AaeXatLxBI9gBaebbnrfifHhDYfgasaacH8akY=wiFfYdH8Gipec8Eeeu0xXdbba9frFj0=OqFfea0dXdd9vqai=hGuQ8kuc9pgc9s8qqaq=dirpe0xb9q8qiLsFr0=vr0=vr0dc8meaabaqaciaacaGaaeqabaqabeGadaaakeaacqWGQbGAdaqhaaWcbaGaemOBa4gabaGaeGymaedaaaaa@308B@ and jn2
 MathType@MTEF@5@5@+=feaafiart1ev1aaatCvAUfKttLearuWrP9MDH5MBPbIqV92AaeXatLxBI9gBaebbnrfifHhDYfgasaacH8akY=wiFfYdH8Gipec8Eeeu0xXdbba9frFj0=OqFfea0dXdd9vqai=hGuQ8kuc9pgc9s8qqaq=dirpe0xb9q8qiLsFr0=vr0=vr0dc8meaabaqaciaacaGaaeqabaqabeGadaaakeaacqWGQbGAdaqhaaWcbaGaemOBa4gabaGaeGOmaidaaaaa@308D@ be their respective indexes in this set. For any haplotype **h **of index *j*, we denote ∏_*j *_as its frequency in the population and ∏_*Q,j *_as the frequency of haplotype (*Q*, **h**) in the population. Conditionally on the vector **∏ **of all haplotype frequencies in the population and assuming Hardy-Weinberg equilibrium, we can now express the probabilities of QTL genotypes given the marker genotypes as follows:

ℒ(x|D,Π)∝∏n=1Ns[φμ+a,σ2(yn)ΠQ,jn1Πjn1ΠQ,jn2Πjn2+φμ−a,σ2(yn)Πq,jn1Πjn1Πq,jn2Πjn2+φμ+d,σ2(yn)(Πq,jn1Πjn1ΠQ,jn2Πjn2+ΠQ,jn1Πjn1Πq,jn2Πjn2)]     (2)
 MathType@MTEF@5@5@+=feaafiart1ev1aaatCvAUfKttLearuWrP9MDH5MBPbIqV92AaeXatLxBI9gBamrtHrhAL1wy0L2yHvtyaeHbnfgDOvwBHrxAJfwnaebbnrfifHhDYfgasaacH8akY=wiFfYdH8Gipec8Eeeu0xXdbba9frFj0=OqFfea0dXdd9vqai=hGuQ8kuc9pgc9s8qqaq=dirpe0xb9q8qiLsFr0=vr0=vr0dc8meaabaqaciaacaGaaeqabaWaaeGaeaaakeaafaqaaeGadaaabaacdaGae8NeHWKaeiikaGIaemiEaGNaeiiFaWNae83aXtKaeiilaWIaeuiOdaLaeiykaKcabaGaeyyhIulabaWaaebCaeaadaWabaqaaGGaciab+z8aMnaaBaaaleaacqGF8oqBcqGHRaWkcqWGHbqycqGGSaalcqGFdpWCdaahaaadbeqaaiabikdaYaaaaSqabaGccqGGOaakcqWG5bqEdaWgaaWcbaGaemOBa4gabeaakiabcMcaPmaalaaabaGaeuiOda1aaSbaaSqaaiabdgfarjabcYcaSiabdQgaQnaaDaaameaacqWGUbGBaeaacqaIXaqmaaaaleqaaaGcbaGaeuiOda1aaSbaaSqaaiabdQgaQnaaDaaameaacqWGUbGBaeaacqaIXaqmaaaaleqaaaaakmaalaaabaGaeuiOda1aaSbaaSqaaiabdgfarjabcYcaSiabdQgaQnaaDaaameaacqWGUbGBaeaacqaIYaGmaaaaleqaaaGcbaGaeuiOda1aaSbaaSqaaiabdQgaQnaaDaaameaacqWGUbGBaeaacqaIYaGmaaaaleqaaaaakiabgUcaRiab+z8aMnaaBaaaleaacqGF8oqBcqGFsislcqWGHbqycqGGSaalcqGFdpWCdaahaaadbeqaaiabikdaYaaaaSqabaGccqGGOaakcqWG5bqEdaWgaaWcbaGaemOBa4gabeaakiabcMcaPaGaay5waaWaaSaaaeaacqqHGoaudaWgaaWcbaGaemyCaeNaeiilaWIaemOAaO2aa0baaWqaaiabd6gaUbqaaiabigdaXaaaaSqabaaakeaacqqHGoaudaWgaaWcbaGaemOAaO2aa0baaWqaaiabd6gaUbqaaiabigdaXaaaaSqabaaaaOWaaSaaaeaacqqHGoaudaWgaaWcbaGaemyCaeNaeiilaWIaemOAaO2aa0baaWqaaiabd6gaUbqaaiabikdaYaaaaSqabaaakeaacqqHGoaudaWgaaWcbaGaemOAaO2aa0baaWqaaiabd6gaUbqaaiabikdaYaaaaSqabaaaaaqaaiabd6gaUjabg2da9iabigdaXaqaaiabd6eaonaaBaaameaacqWGZbWCaeqaaaqdcqGHpis1aaGcbaaabaGaey4kaScabaWaamGaaeaacqGFgpGzdaWgaaWcbaGae4hVd0Maey4kaSIaemizaqMaeiilaWIae43Wdm3aaWbaaWqabeaacqaIYaGmaaaaleqaaOGaeiikaGIaemyEaK3aaSbaaSqaaiabd6gaUbqabaGccqGGPaqkcqGGOaakdaWcaaqaaiabfc6aqnaaBaaaleaacqWGXbqCcqGGSaalcqWGQbGAdaqhaaadbaGaemOBa4gabaGaeGymaedaaaWcbeaaaOqaaiabfc6aqnaaBaaaleaacqWGQbGAdaqhaaadbaGaemOBa4gabaGaeGymaedaaaWcbeaaaaGcdaWcaaqaaiabfc6aqnaaBaaaleaacqWGrbqucqGGSaalcqWGQbGAdaqhaaadbaGaemOBa4gabaGaeGOmaidaaaWcbeaaaOqaaiabfc6aqnaaBaaaleaacqWGQbGAdaqhaaadbaGaemOBa4gabaGaeGOmaidaaaWcbeaaaaGccqGHRaWkdaWcaaqaaiabfc6aqnaaBaaaleaacqWGrbqucqGGSaalcqWGQbGAdaqhaaadbaGaemOBa4gabaGaeGymaedaaaWcbeaaaOqaaiabfc6aqnaaBaaaleaacqWGQbGAdaqhaaadbaGaemOBa4gabaGaeGymaedaaaWcbeaaaaGcdaWcaaqaaiabfc6aqnaaBaaaleaacqWGXbqCcqGGSaalcqWGQbGAdaqhaaadbaGaemOBa4gabaGaeGOmaidaaaWcbeaaaOqaaiabfc6aqnaaBaaaleaacqWGQbGAdaqhaaadbaGaemOBa4gabaGaeGOmaidaaaWcbeaaaaGccqGGPaqkaiaaw2faaaaacaWLjaGaaCzcamaabmaabaGaeGOmaidacaGLOaGaayzkaaaaaa@EB07@

However, the haplotype frequencies in the population are random variables evolving stochastically along generations, and their values at the time that the data are sampled are unknown. Thus the true likelihood is

ℒ(x|D)=E[ℒ(x|D,Π)]     (3)
 MathType@MTEF@5@5@+=feaafiart1ev1aaatCvAUfKttLearuWrP9MDH5MBPbIqV92AaeXatLxBI9gBamrtHrhAL1wy0L2yHvtyaeHbnfgDOvwBHrxAJfwnaebbnrfifHhDYfgasaacH8akY=wiFfYdH8Gipec8Eeeu0xXdbba9frFj0=OqFfea0dXdd9vqai=hGuQ8kuc9pgc9s8qqaq=dirpe0xb9q8qiLsFr0=vr0=vr0dc8meaabaqaciaacaGaaeqabaWaaeGaeaaakeaaimaacqWFsectcqGGOaakcqWG4baEcqGG8baFcqWFdeprcqGGPaqkcqGH9aqpt0uy0HwzTfgDPnwy3aqeh0uy0HwzTfgDPnwy3aacfaGae4hHWxKaei4waSLae8NeHWKaeiikaGIaemiEaGNaeiiFaWNae83aXtKaeiilaWIaeuiOdaLaeiykaKIaeiyxa0LaaCzcaiaaxMaadaqadaqaaiabiodaZaGaayjkaiaawMcaaaaa@5A86@

where the expected value is taken over the probability distribution of haplotype frequencies in the population. This distribution depends on parameters such as the effective population size and the recombination rates between loci, and is specified by mathematical models of population genetics. The general idea of computing the likelihood conditionally on haplotype frequencies in the population and then taking the expected value was first proposed in [29], and was subsequently used in [10] and [8]. However, all these papers were dealing with dichotomous disease traits for which the form of the likelihood was quite different.

### Approximating the likelihood

Under classical models of population genetics, the likelihood function defined by (2) and (3) cannot be easily calculated, and so approximations are necessary. A natural approach is to estimate (3) using a Monte Carlo method, simulating a large number of population replicates for one marker and one disease gene [8]. Unfortunately this approach is very time consuming. In fact, a huge proportion of replicates have to be dropped because the allele frequencies at the final generation are not in good agreement with the ones observed in the sample. A more direct way of computing (3) is to approximate the overall expected value by a expected values; i.e.,

ℒ(x|D)≈∏n=1Ns[φμ−a,σ2(yn)E[Πq,jn1]E[Πjn1]E[Πq,jn2]E[Πjn2]+φμ+a,σ2(yn)E[ΠQ,jn1]E[Πjn1]E[ΠQ,jn2]E[Πjn2]+φμ+d,σ2(yn)(E[Πq,jn1]E[Πjn1]E[ΠQ,jn2]E[Πjn2]+E[ΠQ,jn1]E[Πjn1]E[Πq,jn2]E[Πjn2])]     (4)
 MathType@MTEF@5@5@+=feaafiart1ev1aaatCvAUfKttLearuWrP9MDH5MBPbIqV92AaeXatLxBI9gBamrtHrhAL1wy0L2yHvtyaeHbnfgDOvwBHrxAJfwnaebbnrfifHhDYfgasaacH8akY=wiFfYdH8Gipec8Eeeu0xXdbba9frFj0=OqFfea0dXdd9vqai=hGuQ8kuc9pgc9s8qqaq=dirpe0xb9q8qiLsFr0=vr0=vr0dc8meaabaqaciaacaGaaeqabaWaaeGaeaaakeaafaqabeGadaaabaacdaGae8NeHWKaeiikaGIaemiEaGNaeiiFaWNae83aXtKaeiykaKcabaGaeyisISlabaWaaebCaeaadaWabaqaaGGaciab+z8aMnaaBaaaleaacqGF8oqBcqGHsislcqWGHbqycqGGSaalcqGFdpWCdaahaaadbeqaaiabikdaYaaaaSqabaGccqGGOaakcqWG5bqEdaWgaaWcbaGaemOBa4gabeaakiabcMcaPaGaay5waaWaaSaaaeaat0uy0HwzTfgDPnwy3aqeh0uy0HwzTfgDPnwy3aacfaGae0hHWxKaei4waSLaeuiOda1aaSbaaSqaaiabdghaXjabcYcaSiabdQgaQnaaDaaameaacqWGUbGBaeaacqaIXaqmaaaaleqaaOGaeiyxa0fabaGae0hHWxKaei4waSLaeuiOda1aaSbaaSqaaiabdQgaQnaaDaaameaacqWGUbGBaeaacqaIXaqmaaaaleqaaOGaeiyxa0faaaWcbaGaemOBa4Maeyypa0JaeGymaedabaGaemOta40aaSbaaWqaaiabdohaZbqabaaaniabg+GivdGcdaWcaaqaaiab9ri8fjabcUfaBjabfc6aqnaaBaaaleaacqWGXbqCcqGGSaalcqWGQbGAdaqhaaadbaGaemOBa4gabaGaeGOmaidaaaWcbeaakiabc2faDbqaaiab9ri8fjabcUfaBjabfc6aqnaaBaaaleaacqWGQbGAdaqhaaadbaGaemOBa4gabaGaeGOmaidaaaWcbeaakiabc2faDbaacqGHRaWkcqGFgpGzdaWgaaWcbaGae4hVd0Maey4kaSIaemyyaeMaeiilaWIae43Wdm3aaWbaaWqabeaacqaIYaGmaaaaleqaaOGaeiikaGIaemyEaK3aaSbaaSqaaiabd6gaUbqabaGccqGGPaqkdaWcaaqaaiab9ri8fjabcUfaBjabfc6aqnaaBaaaleaacqWGrbqucqGGSaalcqWGQbGAdaqhaaadbaGaemOBa4gabaGaeGymaedaaaWcbeaakiabc2faDbqaaiab9ri8fjabcUfaBjabfc6aqnaaBaaaleaacqWGQbGAdaqhaaadbaGaemOBa4gabaGaeGymaedaaaWcbeaakiabc2faDbaadaWcaaqaaiab9ri8fjabcUfaBjabfc6aqnaaBaaaleaacqWGrbqucqGGSaalcqWGQbGAdaqhaaadbaGaemOBa4gabaGaeGOmaidaaaWcbeaakiabc2faDbqaaiab9ri8fjabcUfaBjabfc6aqnaaBaaaleaacqWGQbGAdaqhaaadbaGaemOBa4gabaGaeGOmaidaaaWcbeaakiabc2faDbaaaeaaaeaacqGHRaWkaeaadaWacaqaaiab+z8aMnaaBaaaleaacqGF8oqBcqGHRaWkcqWGKbazcqGGSaalcqGFdpWCdaahaaadbeqaaiabikdaYaaaaSqabaGccqGGOaakcqWG5bqEdaWgaaWcbaGaemOBa4gabeaakiabcMcaPiabcIcaOmaalaaabaGae0hHWxKaei4waSLaeuiOda1aaSbaaSqaaiabdghaXjabcYcaSiabdQgaQnaaDaaameaacqWGUbGBaeaacqaIXaqmaaaaleqaaOGaeiyxa0fabaGae0hHWxKaei4waSLaeuiOda1aaSbaaSqaaiabdQgaQnaaDaaameaacqWGUbGBaeaacqaIXaqmaaaaleqaaOGaeiyxa0faamaalaaabaGae0hHWxKaei4waSLaeuiOda1aaSbaaSqaaiabdgfarjabcYcaSiabdQgaQnaaDaaameaacqWGUbGBaeaacqaIYaGmaaaaleqaaOGaeiyxa0fabaGae0hHWxKaei4waSLaeuiOda1aaSbaaSqaaiabdQgaQnaaDaaameaacqWGUbGBaeaacqaIYaGmaaaaleqaaOGaeiyxa0faaiabgUcaRmaalaaabaGae0hHWxKaei4waSLaeuiOda1aaSbaaSqaaiabdgfarjabcYcaSiabdQgaQnaaDaaameaacqWGUbGBaeaacqaIXaqmaaaaleqaaOGaeiyxa0fabaGae0hHWxKaei4waSLaeuiOda1aaSbaaSqaaiabdQgaQnaaDaaameaacqWGUbGBaeaacqaIXaqmaaaaleqaaOGaeiyxa0faamaalaaabaGae0hHWxKaei4waSLaeuiOda1aaSbaaSqaaiabdghaXjabcYcaSiabdQgaQnaaDaaameaacqWGUbGBaeaacqaIYaGmaaaaleqaaOGaeiyxa0fabaGae0hHWxKaei4waSLaeuiOda1aaSbaaSqaaiabdQgaQnaaDaaameaacqWGUbGBaeaacqaIYaGmaaaaleqaaOGaeiyxa0faaiabcMcaPaGaayzxaaaaaiaaxMaacaWLjaWaaeWaaeaacqaI0aanaiaawIcacaGLPaaaaaa@3E1E@

As a consequence of Taylor's expansion and convergence in probability of **∏**, (4) can be proved to converge to the true likelihood as the effective population size tends to infinity. Using this formula is equivalent to assuming that the effective population size is infinite, or that changes in haplotype frequencies along generations are deterministic. This approximation can be refined by adding the second term of the Taylor expansion, which involves second moments of haplotype frequencies ∏_*Q*,*j*_. This was done in the context of a single-marker method by Xiong and Guo [10], who concluded that introducing this second-order term did not significantly improve the location estimates. Therefore, in the following sections we focus on methods using only the first-order approximation in (4).

### Mixture model

Using approximation (4), our model can be described as follows. Each phenotype value *Y*_*n *_is randomly drawn from the mixture of three normal distributions: φμ−a,σ2
 MathType@MTEF@5@5@+=feaafiart1ev1aaatCvAUfKttLearuWrP9MDH5MBPbIqV92AaeXatLxBI9gBaebbnrfifHhDYfgasaacH8akY=wiFfYdH8Gipec8Eeeu0xXdbba9frFj0=OqFfea0dXdd9vqai=hGuQ8kuc9pgc9s8qqaq=dirpe0xb9q8qiLsFr0=vr0=vr0dc8meaabaqaciaacaGaaeqabaqabeGadaaakeaaiiGacqWFgpGzdaWgaaWcbaGae8hVd0MaeyOeI0IaemyyaeMaeiilaWIae83Wdm3aaWbaaWqabeaacqaIYaGmaaaaleqaaaaa@364A@, φμ+a,σ2
 MathType@MTEF@5@5@+=feaafiart1ev1aaatCvAUfKttLearuWrP9MDH5MBPbIqV92AaeXatLxBI9gBaebbnrfifHhDYfgasaacH8akY=wiFfYdH8Gipec8Eeeu0xXdbba9frFj0=OqFfea0dXdd9vqai=hGuQ8kuc9pgc9s8qqaq=dirpe0xb9q8qiLsFr0=vr0=vr0dc8meaabaqaciaacaGaaeqabaqabeGadaaakeaaiiGacqWFgpGzdaWgaaWcbaGae8hVd0Maey4kaSIaemyyaeMaeiilaWIae83Wdm3aaWbaaWqabeaacqaIYaGmaaaaleqaaaaa@363F@ and φμ+d,σ2
 MathType@MTEF@5@5@+=feaafiart1ev1aaatCvAUfKttLearuWrP9MDH5MBPbIqV92AaeXatLxBI9gBaebbnrfifHhDYfgasaacH8akY=wiFfYdH8Gipec8Eeeu0xXdbba9frFj0=OqFfea0dXdd9vqai=hGuQ8kuc9pgc9s8qqaq=dirpe0xb9q8qiLsFr0=vr0=vr0dc8meaabaqaciaacaGaaeqabaqabeGadaaakeaaiiGacqWFgpGzdaWgaaWcbaGae8hVd0Maey4kaSIaemizaqMaeiilaWIae83Wdm3aaWbaaWqabeaacqaIYaGmaaaaleqaaaaa@3645@. The probabilities of being drawn from each of these distributions result from the genetic history of the population. They can be derived under a few assumptions on the population model, as illustrated in the following sections. These probabilities depend on the diplotype hn1
 MathType@MTEF@5@5@+=feaafiart1ev1aaatCvAUfKttLearuWrP9MDH5MBPbIqV92AaeXatLxBI9gBaebbnrfifHhDYfgasaacH8akY=wiFfYdH8Gipec8Eeeu0xXdbba9frFj0=OqFfea0dXdd9vqai=hGuQ8kuc9pgc9s8qqaq=dirpe0xb9q8qiLsFr0=vr0=vr0dc8meaabaqaciaacaGaaeqabaqabeGadaaakeaatCvAUfeBSjuyZL2yd9gzLbvyNv2CaeHbwvMCKfMBHbaceeGaa8hAamaaDaaaleaacqWGUbGBaeaacqaIXaqmaaaaaa@3AA3@/hn2
 MathType@MTEF@5@5@+=feaafiart1ev1aaatCvAUfKttLearuWrP9MDH5MBPbIqV92AaeXatLxBI9gBaebbnrfifHhDYfgasaacH8akY=wiFfYdH8Gipec8Eeeu0xXdbba9frFj0=OqFfea0dXdd9vqai=hGuQ8kuc9pgc9s8qqaq=dirpe0xb9q8qiLsFr0=vr0=vr0dc8meaabaqaciaacaGaaeqabaqabeGadaaakeaatCvAUfeBSjuyZL2yd9gzLbvyNv2CaeHbwvMCKfMBHbaceeGaa8hAamaaDaaaleaacqWGUbGBaeaacqaIYaGmaaaaaa@3AA5@. At the first order, our method is thus equivalent to fitting a linear model **Y **= **X***θ *+ *ε*, where **Y **is the vector of phenotype records, *θ *is the vector of diplotype effects, *ε *is a vector of independent random noises with variance *σ*^2 ^and **X **is a design matrix of size *N*_*s *_× *D*, *D *being the number of diplotypes in the population. Each component of *θ *is a known function of a small number of population parameters which model the LD creation and the evolution process of the population. Each component of *θ *is also supposed to fit the phenotype mean observed for one particular diplotype, so that each diplotype provides one equation. Our aim is to identify the population parameter values that are optimal with respect to the whole set of equations.

### Using marker information

The simultaneous use of more markers should increase the accuracy of the QTL location because the past recombination events can be identified more precisely. However, increasing the number of markers makes the computation of haplotype frequency distribution – and consequently of the likelihood function in (4) -more complex. We previously [18] provided two methods for fine mapping of quantitative traits. The first one was a single-marker method: for each position *x *on the map, only one marker was considered and the expected haplotype frequencies E
 MathType@MTEF@5@5@+=feaafiart1ev1aaatCvAUfKttLearuWrP9MDH5MBPbIqV92AaeXatLxBI9gBamrtHrhAL1wy0L2yHvtyaeHbnfgDOvwBHrxAJfwnaebbnrfifHhDYfgasaacH8akY=wiFfYdH8Gipec8Eeeu0xXdbba9frFj0=OqFfea0dXdd9vqai=hGuQ8kuc9pgc9s8qqaq=dirpe0xb9q8qiLsFr0=vr0=vr0dc8meaabaqaciaacaGaaeqabaWaaeGaeaaakeaat0uy0HwzTfgDPnwy3aqeh0uy0HwzTfgDPnwy3aacfaGae8hHWxeaaa@41FF@[∏_*Q*,*i*_] and E
 MathType@MTEF@5@5@+=feaafiart1ev1aaatCvAUfKttLearuWrP9MDH5MBPbIqV92AaeXatLxBI9gBamrtHrhAL1wy0L2yHvtyaeHbnfgDOvwBHrxAJfwnaebbnrfifHhDYfgasaacH8akY=wiFfYdH8Gipec8Eeeu0xXdbba9frFj0=OqFfea0dXdd9vqai=hGuQ8kuc9pgc9s8qqaq=dirpe0xb9q8qiLsFr0=vr0=vr0dc8meaabaqaciaacaGaaeqabaWaaeGaeaaakeaat0uy0HwzTfgDPnwy3aqeh0uy0HwzTfgDPnwy3aacfaGae8hHWxeaaa@41FF@[∏_*q*,*i*_] were expressed for every allele *i *of this marker as a function of the allele frequencies, the time *t *since the initial creation of LD, the recombination rate *c *between the QTL and the marker locus, the allele initially associated with the mutation *Q*, and a heterogeneity parameter *α *that is described in more detail below. Equation (4) could thus be computed. With only one marker, parameters *t*, *c *and *α *could not be estimated independently of each others so they were combined into a single parameter *λ *= *α*(1 - *c*)^*t*^. The second method was a composite likelihood method that used the set of *L *closest markers at each position whilst assuming that these markers were associated with the QTL independently of each other:

ℒ(x|D)=∏l=1Lℒℓ(x|D)
 MathType@MTEF@5@5@+=feaafiart1ev1aaatCvAUfKttLearuWrP9MDH5MBPbIqV92AaeXatLxBI9gBamrtHrhAL1wy0L2yHvtyaeHbnfgDOvwBHrxAJfwnaebbnrfifHhDYfgasaacH8akY=wiFfYdH8Gipec8Eeeu0xXdbba9frFj0=OqFfea0dXdd9vqai=hGuQ8kuc9pgc9s8qqaq=dirpe0xb9q8qiLsFr0=vr0=vr0dc8meaabaqaciaacaGaaeqabaWaaeGaeaaakeaaimaacqWFsectcqqGOaakcqqG4baEcqqG8baFcqWFdeprcqGGPaqkcqGH9aqpdaqeWbqaaiab=jrimnaaBaaaleaacqWFtecBaeqaaaqaaiabdYgaSjabg2da9iabigdaXaqaaiabdYeambqdcqGHpis1aOGaeiikaGIaemiEaGNaeiiFaWNae83aXtKaeiykaKcaaa@4E3D@

where ℒ
 MathType@MTEF@5@5@+=feaafiart1ev1aaatCvAUfKttLearuWrP9MDH5MBPbIqV92AaeXatLxBI9gBamrtHrhAL1wy0L2yHvtyaeHbnfgDOvwBHrxAJfwnaebbnrfifHhDYfgasaacH8akY=wiFfYdH8Gipec8Eeeu0xXdbba9frFj0=OqFfea0dXdd9vqai=hGuQ8kuc9pgc9s8qqaq=dirpe0xb9q8qiLsFr0=vr0=vr0dc8meaabaqaciaacaGaaeqabaWaaeGaeaaakeaaimaacqWFsectaaa@376E@_*l*_(*x *| D
 MathType@MTEF@5@5@+=feaafiart1ev1aaatCvAUfKttLearuWrP9MDH5MBPbIqV92AaeXatLxBI9gBamrtHrhAL1wy0L2yHvtyaeHbnfgDOvwBHrxAJfwnaebbnrfifHhDYfgasaacH8akY=wiFfYdH8Gipec8Eeeu0xXdbba9frFj0=OqFfea0dXdd9vqai=hGuQ8kuc9pgc9s8qqaq=dirpe0xb9q8qiLsFr0=vr0=vr0dc8meaabaqaciaacaGaaeqabaWaaeGaeaaakeaaimaacqWFdepraaa@3827@) denotes the single-marker likelihood function for the *l*th marker.

The above assumption of independence is clearly violated when markers are linked. To account for a correlation between close loci, Xiong and Guo [10] determined an expression for the expected frequency of haplotypes with one disease gene and two markers. They computed the likelihood function (4) using – at each postulated position of the disease locus – the information from the two flanking markers. Their method takes into account recurrent mutations and population growth since the initial creation of LD. For several experimental data sets, Xiong and Guo showed that their method provided better estimations than those in [8] and [9]. However, their method is based on the assumption that the allele causing the disease is rare, which allows the haplotype frequencies in the healthy population to be modeled as a deterministic process and thus simplifies the derivations.

The above assumption is not appropriate when dealing with QTLs. Consequently, we extended the derivations in [10] to the general case where all haplotype frequencies are random variables following the three-locus Wright-Fisher model. The allele frequencies at markers are still assumed to be deterministic, time invariant, and in equilibrium in the sense that if *i*_1 _and *i*_2 _respectively denote alleles of the left- and right-side markers, ∏i1,i2=∏i1∏i2
MathType@MTEF@5@5@+=feaafiart1ev1aaatCvAUfKttLearuWrP9MDH5MBPbIqV92AaeXatLxBI9gBaebbnrfifHhDYfgasaacH8akY=wiFfYdH8Gipec8Eeeu0xXdbba9frFj0=OqFfea0dXdd9vqai=hGuQ8kuc9pgc9s8qqaq=dirpe0xb9q8qiLsFr0=vr0=vr0dc8meaabaqaciaacaGaaeqabaqabeGadaaakeaadaqeqaqaaiabg2da9maarababaWaaebeaeaaaSqaaiabdMgaPnaaBaaameaacqaIYaGmaeqaaaWcbeqdcqGHpis1aaWcbaGaemyAaK2aaSbaaWqaaiabigdaXaqabaaaleqaniabg+GivdaaleaacqWGPbqAdaWgaaadbaGaeGymaedabeaaliabcYcaSiabdMgaPnaaBaaameaacqaIYaGmaeqaaaWcbeqdcqGHpis1aaaa@3E18@. We proved that the expected frequency of haplotype (*i*_1_, *Q*, *i*_2_) after *t *generations is given by

E[∏i1,Q,i2(t)]=∏Q(0)∏i1∏i2+(1−c1)t(∏i1,Q(0)−∏Q(0)∏i1)∏i2+(1−c2)t(∏Q,i2(0)−∏Q(0)∏i2)∏i1+(1−c1)t(1−c2)t(∏i1,Q,i2(0)−∏i1,Q(0)∏i2−∏Q,i2(0)∏i1+∏Q(0)∏i1∏i2)     (5)
MathType@MTEF@5@5@+=feaafiart1ev1aaatCvAUfKttLearuWrP9MDH5MBPbIqV92AaeXatLxBI9gBamrtHrhAL1wy0L2yHvtyaeHbnfgDOvwBHrxAJfwnaebbnrfifHhDYfgasaacH8akY=wiFfYdH8Gipec8Eeeu0xXdbba9frFj0=OqFfea0dXdd9vqai=hGuQ8kuc9pgc9s8qqaq=dirpe0xb9q8qiLsFr0=vr0=vr0dc8meaabaqaciaacaGaaeqabaWaaeGaeaaakeaafaqaaeGadaaabaGaeeyrauKaee4waS1aaebeaeaacqGGOaakcqWG0baDcqGGPaqkcqGGDbqxaSqaaiabbMgaPnaaBaaameaacqqGXaqmaeqaaSGaeiilaWIaemyuaeLaeiilaWIaemyAaK2aaSbaaWqaaiabikdaYaqabaaaleqaniabg+GivdaakeaacqGH9aqpaeaadaqeqaqaaiabcIcaOiabicdaWiabcMcaPaWcbaGaemyuaefabeqdcqGHpis1aOWaaebeaeaadaqeqaqaaiabgUcaRiabcIcaOiabigdaXiabgkHiTiabdogaJnaaBaaaleaacqaIXaqmaeqaaOGaeiykaKYaaWbaaSqabeaacqWG0baDaaGccqGGOaakdaqeqaqaaiabcIcaOiabicdaWiabcMcaPaWcbaGaemyAaK2aaSbaaWqaaiabigdaXaqabaWccqGGSaalcqWGrbquaeqaniabg+GivdGccqGHsisldaqeqaqaaiabcIcaOiabicdaWiabcMcaPaWcbaGaemyuaefabeqdcqGHpis1aOWaaebeaeaacqGGPaqkdaqeqaqaaiabgUcaRiabcIcaOiabigdaXiabgkHiTiabdogaJnaaBaaaleaacqaIYaGmaeqaaOGaeiykaKYaaWbaaSqabeaacqWG0baDaaGccqGGOaakdaqeqaqaaiabcIcaOiabicdaWiabcMcaPiabgkHiTmaarababaGaeiikaGIaeGimaaJaeiykaKcaleaacqWGrbquaeqaniabg+GivdGcdaqeqaqaaiabcMcaPmaarababaaaleaacqWGPbqAdaWgaaadbaGaeGymaedabeaaaSqab0Gaey4dIunaaSqaaiabdMgaPnaaBaaameaacqaIYaGmaeqaaaWcbeqdcqGHpis1aaWcbaGaemyuaeLaeiilaWIaemyAaK2aaSbaaWqaaiabikdaYaqabaaaleqaniabg+GivdaaleaacqWGPbqAdaWgaaadbaGaeGOmaidabeaaaSqab0Gaey4dIunaaSqaaiabdMgaPnaaBaaameaacqaIXaqmaeqaaaWcbeqdcqGHpis1aaWcbaGaemyAaK2aaSbaaWqaaiabikdaYaqabaaaleqaniabg+GivdaaleaacqWGPbqAdaWgaaadbaGaeGymaedabeaaaSqab0Gaey4dIunaaOqaaaqaaiabbUcaRaqaaiabbIcaOiabbgdaXiabgkHiTiabdogaJnaaBaaaleaacqaIXaqmaeqaaOGaeiykaKYaaWbaaSqabeaacqWG0baDaaGccqGGOaakcqaIXaqmcqGHsislcqWGJbWydaWgaaWcbaGaeGOmaidabeaakiabcMcaPmaaCaaaleqabaGaemiDaqhaaOGaeiikaGYaaebeaeaacqGGOaakcqaIWaamcqGGPaqkaSqaaiabdMgaPnaaBaaameaacqaIXaqmaeqaaSGaeiilaWIaemyuaeLaeiilaWIaemyAaK2aaSbaaWqaaiabikdaYaqabaaaleqaniabg+GivdGccqGHsisldaqeqaqaaiabcIcaOiabicdaWiabcMcaPaWcbaGaemyAaK2aaSbaaWqaaiabigdaXiabcYcaSiabdgfarbqabaaaleqaniabg+GivdGcdaqeqaqaaiabgkHiTmaarababaGaeiikaGIaeGimaaJaeiykaKYaaebeaeaacqGHRaWkdaqeqaqaaiabcIcaOiabicdaWiabcMcaPaWcbaGaemyuaefabeqdcqGHpis1aOWaaebeaeaadaqeqaqaaiabcMcaPaWcbaGaemyAaK2aaSbaaWqaaiabikdaYaqabaaaleqaniabg+GivdaaleaacqWGPbqAdaWgaaadbaGaeGymaedabeaaaSqab0Gaey4dIunaaSqaaiabdMgaPnaaBaaameaacqaIXaqmaeqaaaWcbeqdcqGHpis1aaWcbaGaemyuaeLaeiilaWIaemyAaK2aaSbaaWqaaiabikdaYaqabaaaleqaniabg+GivdaaleaacqWGPbqAdaWgaaadbaGaeGOmaidabeaaaSqab0Gaey4dIunaaaGccaWLjaGaaCzcamaabmaabaGaeGynaudacaGLOaGaayzkaaaaaa@E941@

where *c*_1 _and *c*_2 _respectively denote the recombination rates with the left- and right-side markers, and ∏i1,Q,i2(0),∏i1,Q(0),∏Q,i2(0)
MathType@MTEF@5@5@+=feaafiart1ev1aaatCvAUfKttLearuWrP9MDH5MBPbIqV92AaeXatLxBI9gBaebbnrfifHhDYfgasaacH8akY=wiFfYdH8Gipec8Eeeu0xXdbba9frFj0=OqFfea0dXdd9vqai=hGuQ8kuc9pgc9s8qqaq=dirpe0xb9q8qiLsFr0=vr0=vr0dc8meaabaqaciaacaGaaeqabaqabeGadaaakeaadaqeqaqaaiabcIcaOiabicdaWiabcMcaPiabcYcaSmaarababaGaeiikaGIaeGimaaJaeiykaKcaleaacqWGPbqAdaWgaaadbaGaeGymaedabeaaliabcYcaSiabdgfarbqab0Gaey4dIunakiabcYcaSmaarababaGaeiikaGIaeGimaaJaeiykaKcaleaacqWGrbqucqGGSaalcqWGPbqAdaWgaaadbaGaeGOmaidabeaaaSqab0Gaey4dIunaaSqaaiabdMgaPnaaBaaameaacqaIXaqmaeqaaSGaeiilaWIaemyuaeLaeiilaWIaemyAaK2aaSbaaWqaaiabikdaYaqabaaaleqaniabg+Givdaaaa@4CDD@, and ∏_*Q*_(0) are the frequencies of haplotypes (*i*_1_, *Q*, *i*_2_), (*i*_1_, *Q*), and (*Q*, *i*_2_), and allele *Q *at generation 0, respectively. The derivation of this formula is given in the Appendix. At each postulated location *x*, *c*_1 _and *c*_2 _are deduced from the marker map and the expected value (5) can be used to compute the likelihood (4)

### Initial creation of LD

Our method relies on the assumption that the haplotype frequencies in the population were in equilibrium until a genetic or demographic event suddenly created LD between the QTL and a unique marker haplotype at time 0. Classical examples of such events are the introduction of a favorable allele *Q *into an isolated population, by mutation or migration. After this event, haplotype frequencies evolve along generations as described by (5) until the present generation denoted as *t*.

This model allows us to reduce the number of parameters used to describe haplotype frequencies at time 0. Indeed, following [9] and [10], we introduce a heterogeneity parameter *α *in addition to allele frequencies ∏il
MathType@MTEF@5@5@+=feaafiart1ev1aaatCvAUfKttLearuWrP9MDH5MBPbIqV92AaeXatLxBI9gBaebbnrfifHhDYfgasaacH8akY=wiFfYdH8Gipec8Eeeu0xXdbba9frFj0=OqFfea0dXdd9vqai=hGuQ8kuc9pgc9s8qqaq=dirpe0xb9q8qiLsFr0=vr0=vr0dc8meaabaqaciaacaGaaeqabaqabeGadaaakeaadaqeqaqaaaWcbaGaemyAaK2aaSbaaWqaaiabdYgaSbqabaaaleqaniabg+Givdaaaa@3172@, ∏i2
MathType@MTEF@5@5@+=feaafiart1ev1aaatCvAUfeBSjuyZL2yd9gzLbvyNv2CaerbwvMCKfMBHbqedmvETj2BSbqee0evGueE0jxyaibaieYdOi=BH8vipeYdI8qiW7rqqrFfpeea0xe9Lq=Jc9vqaqpepm0xbbG8FasPYRqj0=yi0lXdbba9pGe9qqFf0dXdHuk9fr=xfr=xfrpiWZqaaeaabiGaaiaacaqabeaabeqacmaaaOqaamaarababaaaleaacaWGPbWaaSbaaWqaaiaaikdaaeqaaaWcbeqdcqGHpis1aaaa@3376@, and ∏_*Q*_(0). This parameter represents the proportion of new copies of allele *Q *introduced at time 0 into the population. Note that *α *= 1 if *Q *did not exist previously in the population. Assuming that new alleles *Q *are associated with allele 1 of both markers, the initial frequencies of (5) can be expressed as

∏i1,Q(0)=(1−α)∏i1∏Q(0)+α∏Q(0)δi1=1∏Q,i2(0)=(1−α)∏i2∏Q(0)+α∏Q(0)δi2=1∏i1,Q,i2(0)=(1−α)∏i1∏i2∏Q(0)+α∏Q(0)δi1=1δi2=1
MathType@MTEF@5@5@+=feaafiart1ev1aaatCvAUfKttLearuWrP9MDH5MBPbIqV92AaeXatLxBI9gBaebbnrfifHhDYfgasaacH8akY=wiFfYdH8Gipec8Eeeu0xXdbba9frFj0=OqFfea0dXdd9vqai=hGuQ8kuc9pgc9s8qqaq=dirpe0xb9q8qiLsFr0=vr0=vr0dc8meaabaqaciaacaGaaeqabaqabeGadaaakqaaeeqaamaarababaGaeiikaGIaeGimaaJaeiykaKcaleaacqWGPbqAdaWgaaadbaGaeGymaedabeaaliabcYcaSiabdgfarbqab0Gaey4dIunakiabg2da9iabcIcaOiabigdaXiabgkHiTGGaciab=f7aHjabcMcaPmaarababaWaaebeaeaacqGGOaakcqaIWaamcqGGPaqkaSqaaiabdgfarbqab0Gaey4dIunaaSqaaiabdMgaPnaaBaaameaacqaIXaqmaeqaaaWcbeqdcqGHpis1aOGaey4kaSIae8xSde2aaebeaeaacqGGOaakcqaIWaamcqGGPaqkcqWF0oazdaWgaaWcbaGaemyAaK2aaSbaaWqaaiabigdaXaqabaWccqGH9aqpcqaIXaqmaeqaaaqaaiabdgfarbqab0Gaey4dIunaaOqaamaarababaGaeiikaGIaeGimaaJaeiykaKcaleaacqWGrbqucqGGSaalcqWGPbqAdaWgaaadbaGaeGOmaidabeaaaSqab0Gaey4dIunakiabg2da9iabcIcaOiabigdaXiabgkHiTiab=f7aHjabcMcaPmaarababaWaaebeaeaacqGGOaakcqaIWaamcqGGPaqkaSqaaiabdgfarbqab0Gaey4dIunakiabgUcaRiab=f7aHnaarababaGaeiikaGIaeGimaaJaeiykaKIae8hTdq2aaSbaaSqaaiabdMgaPnaaBaaameaacqaIYaGmaeqaaSGaeyypa0JaeGymaedabeaaaeaacqWGrbquaeqaniabg+GivdaaleaacqWGPbqAdaWgaaadbaGaeGOmaidabeaaaSqab0Gaey4dIunaaOqaamaarababaGaeiikaGIaeGimaaJaeiykaKcaleaacqWGPbqAdaWgaaadbaGaeGymaedabeaaliabcYcaSiabdgfarjabcYcaSiabdMgaPnaaBaaameaacqaIYaGmaeqaaaWcbeqdcqGHpis1aOGaeyypa0JaeiikaGIaeGymaeJaeyOeI0Iae8xSdeMaeiykaKYaaebeaeaadaqeqaqaamaarababaGaeiikaGIaeGimaaJaeiykaKcaleaacqWGrbquaeqaniabg+GivdaaleaacqWGPbqAdaWgaaadbaGaeGOmaidabeaaaSqab0Gaey4dIunakiabgUcaRiab=f7aHnaarababaGaeiikaGIaeGimaaJaeiykaKIae8hTdq2aaSbaaSqaaiabdMgaPnaaBaaameaacqaIXaqmaeqaaSGaeyypa0JaeGymaedabeaakiab=r7aKnaaBaaaleaacqWGPbqAdaWgaaadbaGaeGOmaidabeaaliabg2da9iabigdaXaqabaaabaGaemyuaefabeqdcqGHpis1aaWcbaGaemyAaK2aaSbaaWqaaiabigdaXaqabaaaleqaniabg+Givdaaaaa@B182@

where *δ*_*x *= *y *_is the Kronecker delta operator (equal to 1 if *x *= *y *and 0 otherwise).

This model can even be used in a more general context than the introduction of a new allele into an isolated population. Indeed, we know that many of the current isolated populations in both humans and animals [30,31] were initially created by a severe bottleneck in a wider population, implying the underrepresentation of many haplotypes and the over represent at ion of others. After such events, it would not be surprising for an allele of rather low frequency to become associated in the new population with a very small number of marker haplotypes. Our model thus applies to that case, provided that time 0 refers to the creation of the population (while the mutation occurred earlier). Parameter *α *then represents the excess of the overrepresented haplotype including allele *Q*. However, this is only a rough approximation since the favorable allele may in general be associated with more than one haplotype. Many animal breeding populations have also been created by the artificial admixing of two other populations (see [31] for a review), but the amount of LD created between two loci depends on the difference of allele frequencies at these loci between the initial populations. Since this difference is not the same for all loci, there is no reason why a single unique coefficient *α *should be used to model the initial level of association of *Q *with all markers. Consequently, our method appears to be unsuitable for such cases.

### Simulation Results

As outlined above, one fundamental feature of a mapping method is its ability to simultaneously use the information from several markers. We have previously [18] proposed a single-marker method (Tl) and two composite likelihood methods (T2 and T6) to map QTLs using LD. Based on simulation results, our conclusions were that (i) composite likelihood methods provide better location estimates than single-marker methods such as regression analysis or Tl, and (ii) among composite likelihood methods, the one using two markers (T2) generally performs the best.

Starting from these conclusions, we first compare our new method – which we have called HAPim – with T2. While haplotype methods are generally considered to be more accurate than composite likelihood ones, we considered it important to evaluate the exact difference between them, as well as the influence of parameters such as effective population size, marker spacing, and time since the initial creation of LD. We also discuss the behavior of both methods in the presence of incomplete association or phenocopies. We then compare the accuracy of our method with that of the haplotype method in [21]. Both of the following analyses are based on the simulation framework described in the Methods section.

#### Comparison with a composite likelihood method

We first compared HAPim and T2 by reproducing simulation scenarios similar to those in [18]. The QTL was simulated at position 3.6 cM on a 10-cM marker map. Two effective population sizes (*N *= 200 and *N *= 400), two marker-spacing values (0.25 and 2 cM), and both single nucleotide polymorphisms (SNPs) and microsatelites (MSTs) were tested. The time since the initial LD creation was *t *= 100, and no copy of allele *Q *was present in the population before that time, which ensured that complete initial LD was present. The mean square errors (MSEs) of both mapping methods under these various scenarios are given in Table 1. Unsurprisingly, they both performed better with decreasing marker spacing, increasing effective population size and multiallelic markers. However, we were more interested in the influence of parameters on the difference in precision between the methods than on their absolute precisions (which has already been widely studied). Table 1 indicates that the gain from using HAPim is particularly significant with dense maps, irrespective of the marker type and effective population size. This was expected because T2 assumes independence between the QTL-marker associations, which is increasingly violated as the marker spacing decreases.

Table 2 presents the quality of the estimates for all the model parameters using SNP markers, an effective population size of 400, and a marker spacing of 0.25 cM. The QTL location estimate from HAPim was almost unbiased and, as evident in Table 1, more precise than the one from T2. The additive and dominance effects were also very accurately estimated, again better than T2 for the dominance effect. Both methods slightly underestimated heterogeneity parameter *α*, due to it being constrained to be less than 1. The time since the initial creation of LD was very poorly estimated, which is the case with all LD mapping methods [16,21]. However, this does not affect the estimation of other parameters because *t *has little effect on the value of the likelihood function. The ∏_*Q*_(0) estimate is nearly the same for both methods. The large difference from the true value of ∏_*Q*_(0) is due to the simulation procedure that rejects the sample paths leading to the final frequency ∏_*Q*_(*t*) being smaller than 0.05. Using the Wright-Fisher model described in the Appendix, it can be proved that ∏_*Q*_(0) is equal to the expectation of ∏_*Q*_(*t*). Therefore, the empirical mean of ∏_*Q*_(0) over the 500 replicates is actually an estimate of the conditional expected value of ∏_*Q*_(*t*) given that 0.05 ≤ ∏_*Q*_(*t*) and ∏_*Q*_(0) = 0.00125. Using a diffusion approximation of the Wright-Fisher process and the corresponding probability density given in [32], we found that this quantity was equal to 0.105. The empirical mean of ∏_*Q*_(0) is in good agreement with this theoretical value, and the slight remaining bias might come from the selective advantage given to allele *Q *in the first few generations of our simulations, which is not accounted for in the diffusion approximation.

Tables 3, 4, and 5 focus on a marker spacing of 0.125 cM, because the results of Table 1 indicate that the gain from using HAPim was greater with dense maps. We considered only biallelic markers, since in practice MSTs are rarely found with such a density. We investigated the role of (i) effective population size *N *(Table 3), and found that as *N *increases, the MSEs of both methods decrease but the difference between the methods becomes less significant; (ii) sample size (Table 4), and found that for *N *= 400 and *N *= 800, the gain of HAPim over T2 appears to recover since a sample from the population is used instead of the entire population; this gain was always significant, particularly with small samples; and (iii) time since the initial LD creation (Table 5), and found that when this time is small, the accuracy of both methods is limited; it is increased with larger evolution times, in which cases HAPim performed much better than T2; it is well known that short evolution times result in the high LD area extending to many markers around the QTL, which limits the accuracy of LD mapping methods in general.

Elucidating the mechanisms underlying the results of such simulations is extremely difficult, because parameters share complex interactions – increasing a particular parameter may have either a positive or a negative effect on the accuracy, depending on the value of the other parameters. Our model describes the decay in the LD from an initial event. In this context, we know that the accuracy of both LD methods mostly depends on the value of the product *ct *[3], with *ct *≈ 2 being optimal. This may explain the results of Table 5. However, this explanation is only applicable to large values of *N*; for smaller values of *N*, at least two phenomena affect this rule. First, the approximation of the likelihood (3) is worse than with large *N *(but we do not know whether T2 or HAPim is affected the most). Second, the LD created by random drift along generations is no longer negligible, and its amount depends on the product *Nc *[29]. However, Tables 3 and 4 suggest that unless the sample size is very large (which also requires a very large effective population size), it is really worth using HAPim instead of T2. HAPim models the evolution of haplotype frequencies more precisely, which balances the lack of information.

Table 4 also includes, for each effective population size and sample size, the power of HAPim and T2 to detect the QTL. This power was estimated from the same 500 replicates as the MSEs, using an approximate threshold as explained in the Methods section. As expected and observed in [33], the power was greater with greater sample size and with lower effective population size. The power results were also consistant with the MSE results: they revealed an important gain from using HAPim, that decreased as sample size increased. The number of replicates in which the log-likelihood ratio test was higher with HAPim than with T2 ranged from 80% to 90% depending on *N *and *N*_*s*_. In Tables 3 and 5, this proportion was generally lower (even 50% with *t *= 300, Table 5) and the power obtained with both methods was always around 1. However the MSEs were still better with HAPim, which indicates that this method also allows a better discrimination between positions.

To complete our study, we compared the robustness of both methods to more complex evolution scenarios. In the first scenario, LD was initially created in a population in which allele *Q *already existed and was in linkage equilibrium with other markers. Since the degree of the initial association is strongly related to the number of alleles, we included both MST and SNP markers. We took a marker spacing of 0.25 cM and an effective population size *N *= 400, as previously done in Table 1. The results listed in Table 6 indicate that the MSEs were smaller than in the corresponding homogeneity scenario of Table 1, despite that heterogeneity decreased the strength of association between the QTL and marker alleles. This is probably due to the frequency of allele *Q *being higher in the heterogeneity scenario, which increases the percentage of the trait variance explained by the QTL and hence improves the mapping precision. HAPim strongly outperformed T2, particularly for MSTs.

In the second scenario we introduced phenocopies. As in the heterogeneity scenario, we chose *N *= 400, a marker spacing of 0.25 cM, and both SNP and MST markers. The MSEs with this scenario, given in Table 7, were much larger than in the corresponding scenario of Table 1, particularly for SNPs. MSTs are less affected by phenocopies because the number of possible marker haplotypes that can be carried by a "false *Q*" individual is much larger than with SNPs. The risk of the method producing a false-positive error is thus reduced. Using HAPim instead of T2 also reduces this risk, because the allele frequencies at flanking markers are modeled jointly. In this scenario, HAPim clearly outperformed T2.

#### Comparison with other haplotype methods

Modeling the information from haplotypes consisting of more than two markers may improve the precision of location estimates. Therefore, further simulations were carried out to compare our HAPim method with the IBD method of Meuwissen and Goddard [21]. Their method is one of the most classical full-haplotype methods, and the similarity of their genetic model to ours makes the comparison easier than for coalescent-based methods such as in [20,23]. We duplicated the simulation scenarios described in Table 2 in [21]: 50 population replicates with biallelic markers initially at equal frequencies with spacings of 0.25, 0.5, and 1.0 cM, an effective population size and a sample size of *N *= *N*_*s *_= 100, and a time *t *= 100 since the initial mutation. The QTL was in the middle of the chromosome region. In order for the results to be perfectly comparable, the mutant allele was not given a slight selective advantage after the mutation time (in contrast to previous simulation scenarios, as explained in the Methods section). Table 8 presents the distribution of the deviations (in marker intervals) in the QTL location estimates from the correct bracket. The results can be directly compared with those of Table 3 in [21]. A chi-square test of equality between the deviation distributions of HAPim and [21] revealed no significant difference (the smallest *p *value was 0.08), and a t-test on the MSEs of both methods also did not reveal any significant difference.

We also tested our method under the simulation scenarios used by Grapes and colleagues [26,27], who compared single- and two-marker regression analysis with an IBD method very similar to that in [21]. For the same number of markers, the least-square mean absolute differences (LSMDs) between the estimated and the true QTL location were clearly smaller with the IBD full-haplotype method ([26], Table 2), which confirms its superiority. A subsequent study [27] revealed that mapping precision of the IBD method could be increased by using a smaller window of markers (four or six), and that using a window of only two markers provided the same accuracy as using the full haplotype (ten markers). We reproduced these simulation scenarios using the same number of replicates (1000) as they used. The results we obtained with HAPim were similar to the ones given by their IBD method using two-marker haplotypes: LSMDs of 1.36, 0.71, and 0.39 for marker spacings of 1.0, 0.5, and 0.25 cM, respectively.

## Discussion

The present simulation study focused on particular values of model parameters, and hence the revealed good properties of HAPim may not hold for other values. However, we consider that the range of parameter values explored includes most of the situations where LD information can be used efficiently for mapping. For instance, the largest value of *t *we considered was 300 (Table 5), and whilst many favorable mutations are much older than 300 generations, it is very unlikely for a population to satisfy the strong hypotheses of the assumed Wright-Fisher model (e.g., random mating and no migrations) over such a long period. In many cases a strong founder effect occurred quite recently, and this event then corresponds to time 0 in our method. In other situations, we know that recurrent mutations or migrations have occurred continuously in the population and consequently perturbed the LD structure. It is very likely that no method could exploit the LD information for mapping in such cases [30,31].

We consider effective population sizes between 100 and 1600 to be realistic for most breeding populations, where the high level of inbreeding reduces the effective size. The effective size of the isolated human populations typically used in LD studies (e.g., Finnish or Caucasian) is generally around 10,000 [10]. We were not able to study such cases, but extrapolating the results of Table 3 leads to the supposition that there is no difference between T2 and HAPim for such large populations, provided that the marker spacing remains larger than around 0.1 cM. Another specific feature of such isolated human populations is their exponential growth rate. It would be easy to include this in our model, but it would have no effect as long as the first-order approximation of the likelihood (4) is used [10]. Another case that we did not study is that of very dense maps (marker spacing smaller than 0.01 cM). In that case the flanking marker haplotypes probably lose relevant information contained in full haplotypes, and modeling the information from more than two markers may improve the mapping precision. Our method could be extended by replacing – on each side of the QTL – the flanking marker by a flanking haplotype, and then performing the computations exactly as before. The extension is straightforward if we assume linkage equilibrium between all markers, but an increased precision is not guaranteed since background LD is not accounted for. As an alternative to assuming equilibrium, one could model marker allele frequencies along the chromosome as a first-order Markov chain with parameters estimated from the marker data at time *t *[12,13], but it would be more difficult to integrate this change in the derivations given in the Appendix.

The model itself and its hypotheses can be criticized. For example, we assume that the marginal allele frequencies are constant and that markers are in linkage equilibrium; i.e., ∏i1,i2=∏i1∏i2
MathType@MTEF@5@5@+=feaafiart1ev1aaatCvAUfKttLearuWrP9MDH5MBPbIqV92AaeXatLxBI9gBaebbnrfifHhDYfgasaacH8akY=wiFfYdH8Gipec8Eeeu0xXdbba9frFj0=OqFfea0dXdd9vqai=hGuQ8kuc9pgc9s8qqaq=dirpe0xb9q8qiLsFr0=vr0=vr0dc8meaabaqaciaacaGaaeqabaqabeGadaaakeaadaqeqaqaaiabg2da9maarababaWaaebeaeaaaSqaaiabdMgaPnaaBaaameaacqaIYaGmaeqaaaWcbeqdcqGHpis1aaWcbaGaemyAaK2aaSbaaWqaaiabigdaXaqabaaaleqaniabg+GivdaaleaacqWGPbqAdaWgaaadbaGaeGymaedabeaaliabcYcaSiabdMgaPnaaBaaameaacqaIYaGmaeqaaaWcbeqdcqGHpis1aaaa@3E18@. Actually, the expression we obtained for E[∏i1,Q,i2(t)]
MathType@MTEF@5@5@+=feaafiart1ev1aaatCvAUfKttLearuWrP9MDH5MBPbIqV92AaeXatLxBI9gBamrtHrhAL1wy0L2yHvtyaeHbnfgDOvwBHrxAJfwnaebbnrfifHhDYfgasaacH8akY=wiFfYdH8Gipec8Eeeu0xXdbba9frFj0=OqFfea0dXdd9vqai=hGuQ8kuc9pgc9s8qqaq=dirpe0xb9q8qiLsFr0=vr0=vr0dc8meaabaqaciaacaGaaeqabaWaaeGaeaaakeaat0uy0HwzTfgDPnwy3aqeh0uy0HwzTfgDPnwy3aacfaGae8hHWxKaei4waS1aaebeaeaacqGGOaakcqWG0baDcqGGPaqkaSqaaiabdMgaPnaaBaaameaacqaIXaqmaeqaaSGaeiilaWIaemyuaeLaeiilaWIaemyAaK2aaSbaaWqaaiabikdaYaqabaaaleqaniabg+GivdGccqGGDbqxaaa@5171@ would be the same if we only assumed equilibrium at time 0 between markers. Considering only the first moment of haplotype frequencies, as we do in (4), this is the best we can do. Accounting for the LD between markers would thus require consideration of a second-order approximation of the likelihood and of the variances of the haplotype frequencies. This may improve the performance of the method, whereas no improvement was observed in [10]. In our simulations the marker frequencies were not constant and the equilibrium imposed at the first simulated generation was randomly broken by drift in the few generations until the time of the mutation. Thus, at time 0 the markers were not in equilibrium. One other strong approximation of the model is the absence of mutations or selection. While the effect of mutations is often negligible on the short evolution times we are interested in, they could be easily accounted for in the derivations of E[∏i1,Q,i2(t)]
MathType@MTEF@5@5@+=feaafiart1ev1aaatCvAUfKttLearuWrP9MDH5MBPbIqV92AaeXatLxBI9gBamrtHrhAL1wy0L2yHvtyaeHbnfgDOvwBHrxAJfwnaebbnrfifHhDYfgasaacH8akY=wiFfYdH8Gipec8Eeeu0xXdbba9frFj0=OqFfea0dXdd9vqai=hGuQ8kuc9pgc9s8qqaq=dirpe0xb9q8qiLsFr0=vr0=vr0dc8meaabaqaciaacaGaaeqabaWaaeGaeaaakeaat0uy0HwzTfgDPnwy3aqeh0uy0HwzTfgDPnwy3aacfaGae8hHWxKaei4waS1aaebeaeaacqGGOaakcqWG0baDcqGGPaqkaSqaaiabdMgaPnaaBaaameaacqaIXaqmaeqaaSGaeiilaWIaemyuaeLaeiilaWIaemyAaK2aaSbaaWqaaiabikdaYaqabaaaleqaniabg+GivdGccqGGDbqxaaa@5171@ using a stepwise mutation model [34]. Selection advantages for *Q *or *q *would be more difficult to incorporate, because they make the expression of E[∏i1,Q,i2(t+1)]
MathType@MTEF@5@5@+=feaafiart1ev1aaatCvAUfKttLearuWrP9MDH5MBPbIqV92AaeXatLxBI9gBamrtHrhAL1wy0L2yHvtyaeHbnfgDOvwBHrxAJfwnaebbnrfifHhDYfgasaacH8akY=wiFfYdH8Gipec8Eeeu0xXdbba9frFj0=OqFfea0dXdd9vqai=hGuQ8kuc9pgc9s8qqaq=dirpe0xb9q8qiLsFr0=vr0=vr0dc8meaabaqaciaacaGaaeqabaWaaeGaeaaakeaat0uy0HwzTfgDPnwy3aqeh0uy0HwzTfgDPnwy3aacfaGae8hHWxKaei4waS1aaebeaeaacqGGOaakcqWG0baDcqGHRaWkcqaIXaqmcqGGPaqkcqGGDbqxaSqaaiabdMgaPnaaBaaameaacqaIXaqmaeqaaSGaeiilaWIaemyuaeLaeiilaWIaemyAaK2aaSbaaWqaaiabikdaYaqabaaaleqaniabg+Givdaaaa@5339@ (see (8) in Appendix) non linear in **∏**(*t*). Finally, it should be noted that ∏_*Q*_(*t*) was not assumed to be constant in our model; this assumption was made in [10] and criticized in [35].

Knowledge of the haplotypes is required to apply haplotype-based mapping methods including the one described here. In our simulations we used a true set of haplotypes, but in the analysis of real data the haplotypes have to be inferred from the data or using pedigree information. Several algorithms have been proposed in the literature to perform such inferences [36]. Combined advances in both these algorithms and molecular haplotyping methods will enable this question to be solved more efficiently in the future. Moreover, several studies [37,38] have shown that the efficiency of fine mapping methods is not greatly reduced by uncertainty of the haplotype phases. If this did not hold for HAPim, the gain from using this method rather than T2 would be low given that T2 is not affected by the haplotype phases. This should be investigated in the future.

In our simulation study the results obtained with HAPim were similar to the ones given by the IBD method using two-marker haplotypes. Nevertheless, there are fundamental differences between HAPim and the IBD method. First, haplotype effects are modeled as fixed effects in the former and as random effects in the latter. While it is well-known that location parameters are easier to estimate than dispersion parameters, it is not clear whether this has a significant effect on the estimation of the QTL position. Second, the IBD method doesn't include dominance effects, while HAPim handles that very efficiently, as illustrated in Table 2. Third, the time *t *since the initial creation of LD and the effective population size *N *have to be known before using the IBD method. Some simulation results in [21] suggested that the default choice of *t *= 100 and *N *= 100 was almost optimal, whatever the true value of these parameters. However the comparison of tables V and VII in [22] indicates that the IBD matrix with *N *= 1000 is really different from the one with *N *= 100. Thus it is not obvious why the IBD method assuming *N *= 100 should be accurate for a population of actual effective size *N *= 1000. On the other hand, neither *t *nor *N *are required for the use of HAPim. Consequently this method can be used in a wider range of populations. A nice advantage of the IBD method is its ability to deal with haplotypes composed of more than two markers. If used with caution, this can provide more accuracy in location estimates [27]. As explained previously, HAPim could also offer this possibility in the future. At present, the several differences highlighted in this paragraph already justify the interest of this method.

An important purpose of QTL mapping methods is to provide a confidence interval for the QTL location. Classical pedigree linkage analyses have proposed log-odds (LOD)-support intervals [39], similar confidence intervals [40], and bootstrap confidence intervals [41]. The simplicity of the bootstrap technique, its ease of implementation, and the accuracy of the coverage probability makes it an appealing approach to use. In LD mapping methods, the coverage accuracy of the LOD-support interval and the credible interval in the Bayesian framework have been studied only for disease traits [12,23,42]. Simulations have shown that both intervals are either unbiased or only slightly conservative. This issue has not yet been addressed for QTL location. An anticonservative bootstrap confidence interval was obtained when we ran a preliminary single simulation with HAPim, which may indicate that the classical bootstrap scheme we used – sampling with replacement of entire records – did not produce enough variability of the QTL location estimate. Confirmation of this result may indicate that providing a correct confidence interval for the QTL location is a challenging and tricky problem.

Although our two-marker haplotype model was basically designed for unrelated individuals, it can also be used in situations where pedigree information is available. For instance, in studies involving large half-sib families, our model can easily be integrated in the combined LD and linkage mapping method of Farnir and colleagues [19]. In their method, LD information is contained in the probabilities of Table 1 ([19], p. 277). These probabilities were derived under a single-marker model, and could instead be derived under our two-marker model using (5) without changing the rest of the method. However, the use of combined LD and pedigree information appears to be more efficient in designs with many small families than in those with a few large families [43]. Consequently a promising strategy for future QTL mapping studies would be to genotype and phenotype more unrelated individuals and use the parental information (if any is available) to infer the haplotypes. In this context the use of our method could be fruitful.

## Conclusion

We have presented a new method for the fine mapping of QTLs, denoted HAPim. It is a likelihood method, whose originality is in modeling the frequencies of haplotypes comprising one trait locus and two flanking markers. Theoretical derivations under this evolution model avoid the intensive computations required to evaluate the likelihood values at each location.

Our simulations have demonstrated the excellent properties of our method. Over a wide range of parameter values (effective population sizes and sample sizes from 200 to 1600, times since LD creation from 50 to 300 generations, and marker spacings from 0.125 to 2 cM), the MSEs obtained with HAPim were almost always significantly lower than those obtained with composite likelihood method T2. Combined with a previous study [18], these results show that HAPim is more accurate than single-marker methods and composite likelihood methods in general. The power to detect the QTL was also greater with HAPim. With approximately the same parameter values, we observed that HAPim was as accurate as the classical IBD method [21] used with two- or ten-marker haplotypes. It also has several advantages over the IBD method, as the ability to incorporate dominance effects and to deal as easily with any value of *t *or *N*. Finally, our simulations suggested that the use of MSTs is very efficient if the analysis is performed with HAPim: the computing time was longer than with SNPs but was still reasonable, and the estimates were more robust to departures from the assumed model. Given that more and more mapping studies are being designed with SNP, this suggests that close SNPs should be combined into groups of two or three to build pseudo-multiallelic markers that avoid spurious associations.

Our method could be improved in several ways, such as by modeling mutations or LD between markers, and using haplotypes with more than two markers, but it is unclear whether these modifications would increase the precision. Providing confidence intervals – in addition to the point-wise QTL location estimates – will also be an interesting challenge. The continuing advances in genotyping and haplotyping technologies will increase the importance of LD fine mapping methods, even in situations where pedigree information is available.

## Methods

### Likelihood maximization

The description of the model highlights that parameters other than the QTL location *x *have to be estimated: the time *t *since the initial creation of LD, the initial frequency ∏_*Q*_(0) of allele *Q*, the initial associated haplotype *j*, and the heterogeneity parameter *α*. We take the values that satisfy

max⁡x,t∏Q(0),j,α,μ,a,d,σ2ℒ(x,t,∏Q(0),j,α,μ,a,d,σ2|D)
MathType@MTEF@5@5@+=feaafiart1ev1aaatCvAUfeBSjuyZL2yd9gzLbvyNv2CaerbwvMCKfMBHbqedmvETj2BSbWenfgDOvwBHrxAJfwnHbqeg0uy0HwzTfgDPnwy1aqee0evGueE0jxyaibaieYdOi=BH8vipeYdI8qiW7rqqrFfpeea0xe9Lq=Jc9vqaqpepm0xbbG8FasPYRqj0=yi0lXdbba9pGe9qqFf0dXdHuk9fr=xfr=xfrpiWZqaaeaabiGaaiaacaqabeaadaqacqaaaOqaamaaxababaGaciyBaiaacggacaGG4baaleaacaWG4bGaaiilaiaadshadaqeqaqaaiaacIcacaaIWaGaaiykaaadbaGaamyuaaqab4Gaey4dIunaliaacYcacaWGQbGaaiilaGGaciab=f7aHjaacYcacqWF8oqBcaGGSaGaamyyaiaacYcacaWGKbGaaiilaiab=n8aZnaaCaaameqabaGaaGOmaaaaaSqabaacdaGccqGFsectcaGGOaGaamiEaiaacYcacaWG0bGaaiilamaarababaGaaiikaiaaicdacaGGPaaaleaacaWGrbaabeqdcqGHpis1aOGaaiilaiaadQgacaGGSaGae8xSdeMaaiilaiab=X7aTjaacYcacaWGHbGaaiilaiaadsgacaGGSaGae83Wdm3aaWbaaSqabeaacaaIYaaaaOGaaiiFaiab+nq8ejaacMcaaaa@6AFA@

This maximization is carried out numerically using the E04CCF simplex algorithm from the NAG library [44]. Marker allele frequencies ∏il
MathType@MTEF@5@5@+=feaafiart1ev1aaatCvAUfKttLearuWrP9MDH5MBPbIqV92AaeXatLxBI9gBaebbnrfifHhDYfgasaacH8akY=wiFfYdH8Gipec8Eeeu0xXdbba9frFj0=OqFfea0dXdd9vqai=hGuQ8kuc9pgc9s8qqaq=dirpe0xb9q8qiLsFr0=vr0=vr0dc8meaabaqaciaacaGaaeqabaqabeGadaaakeaadaqeqaqaaaWcbaGaemyAaK2aaSbaaWqaaiabdYgaSbqabaaaleqaniabg+Givdaaaa@3172@ and ∏i2
MathType@MTEF@5@5@+=feaafiart1ev1aaatCvAUfeBSjuyZL2yd9gzLbvyNv2CaerbwvMCKfMBHbqedmvETj2BSbqee0evGueE0jxyaibaieYdOi=BH8vipeYdI8qiW7rqqrFfpeea0xe9Lq=Jc9vqaqpepm0xbbG8FasPYRqj0=yi0lXdbba9pGe9qqFf0dXdHuk9fr=xfr=xfrpiWZqaaeaabiGaaiaacaqabeaabeqacmaaaOqaamaarababaaaleaacaWGPbWaaSbaaWqaaiaaikdaaeqaaaWcbeqdcqGHpis1aaaa@3376@ also have to be estimated. We use their empirical frequencies in the sample and thus do not need to include them in the likelihood maximization.

We also tested a homogeneity method where *a *was arbitrarily set to 1. On the basis of simulation results (similar to those presented in this paper), we finally dropped this because it was not as robust as the more general method to departures from the assumed model.

### Simulation procedure

We used forward simulations as outlined in [18,45]. The baseline scenario was as follows. We initially define a population of 2*N *haplotypes with *L *equally spaced markers, either biallelic (SNPs) or multiallelic (MSTs) with five alleles. In both cases, all of the marker alleles have the same frequency and the markers are in linkage equilibrium. Then, each new generation is created by sampling N pairs of haplotypes at random from the current generation and allowing random recombinations within these pairs. The recombination rate for each marker interval is computed using Haldane's mapping function. We let the population evolve for 20 × (*N*/400) generations in order to break the linkage equilibrium between markers with a random drift force that does not depend on the effective population size. At time 0, a mutated allele *Q *is introduced at the QTL location on a single haplotype, and again we let the population evolve as previously. At time *t*, a sample of *N*_*s *_individuals is collected, and phenotypes for the trait are simulated according to the model in (1), with *a *= 1, *d *= 1 (complete dominance) and *σ*^2 ^= 1. In all simulation scenarios but the one reported in Table 3, the sample size *N*_*s *_was equal to the effective population size *N*.

Two extensions of this scenario were also considered. Firstly, some copies of allele *Q *were introduced into the population from the first generation of the simulation, with frequency ∏_*Q*_(0) equal to 0.05 or 0.10. These earlier copies of *Q *were in equilibrium with all markers, so at time 0 the association created between *Q *and one particular marker haplotype was incomplete. Secondly, we allowed the presence of phenocopies; i.e., phenotypes that mimic the phenotype produced by the mutation. To reproduce this effect, a given percentage of the individuals carrying allele *q *(15% or 30%) were randomly drawn in the last generation and were given the same genetic effect as individuals carrying allele *Q*.

In all scenarios, replicates were discarded when fixation occurred for the QTL or any of the markers, or when the final frequency of allele *Q *was less than 0.05 or greater than because rare QTL alleles account for a small proportion of the trait variance and are not of interest in QTL mapping studies. To reduce the number of discarded replicates, the new QTL allele was conferred with a slight selective advantage during a few generations after time 0.

The accuracy of QTL location estimates was evaluated according to the MSE defined as

MSE=1R∑r=1R(x^r−x)2
 MathType@MTEF@5@5@+=feaafiart1ev1aaatCvAUfKttLearuWrP9MDH5MBPbIqV92AaeXatLxBI9gBaebbnrfifHhDYfgasaacH8akY=wiFfYdH8Gipec8Eeeu0xXdbba9frFj0=OqFfea0dXdd9vqai=hGuQ8kuc9pgc9s8qqaq=dirpe0xb9q8qiLsFr0=vr0=vr0dc8meaabaqaciaacaGaaeqabaqabeGadaaakeaacqWGnbqtcqWGtbWucqWGfbqrcqGH9aqpdaWcaaqaaiabigdaXaqaaiabdkfasbaadaaeWbqaaiabcIcaOiqbdIha4zaajaWaaSbaaSqaaiabdkhaYbqabaGccqGHsislcqWG4baEcqGGPaqkdaahaaWcbeqaaiabikdaYaaaaeaacqWGYbGCcqGH9aqpcqaIXaqmaeaacqWGsbGua0GaeyyeIuoaaaa@426E@

where *R *is the number of replicates (equal to 500 unless otherwise specified), x^
 MathType@MTEF@5@5@+=feaafiart1ev1aaatCvAUfKttLearuWrP9MDH5MBPbIqV92AaeXatLxBI9gBaebbnrfifHhDYfgasaacH8akY=wiFfYdH8Gipec8Eeeu0xXdbba9frFj0=OqFfea0dXdd9vqai=hGuQ8kuc9pgc9s8qqaq=dirpe0xb9q8qiLsFr0=vr0=vr0dc8meaabaqaciaacaGaaeqabaqabeGadaaakeaacuWG4baEgaqcaaaa@2E35@_*r *_is the estimated QTL location in the *r*th replicate, and *x *is the true location. The MSE contains information of both the bias and the variance of location estimates. Differences in MSE between methods were tested using paired *t*-tests while assuming normality.

### Power computation

Together with the set of optimal parameter values, HAPim returns the log-likelihood ratio test between the null hypothesis "*a *= *d *= 0" and its alternative. In order to compare the power of T2 and HAPim we computed an approximate threshold for any set of population parameter values (*N*, *t*, marker spacing …). This threshold was obtained as the empirical 0.95 quantile of 500 replicates under the null hypothesis.

## Authors' contributions

SB and JA contributed equally to this work. SB developed the mathematical description and JA wrote the computer programs, and they both were involved in the preparation of the draft manuscript. All authors participated in the design conception, the interpretation of the simulation results, and the elaboration of the manuscript under the leadership of BM.

## Appendix

### Derivation of the formula for E
 MathType@MTEF@5@5@+=feaafiart1ev1aaatCvAUfKttLearuWrP9MDH5MBPbIqV92AaeXatLxBI9gBamrtHrhAL1wy0L2yHvtyaeHbnfgDOvwBHrxAJfwnaebbnrfifHhDYfgasaacH8akY=wiFfYdH8Gipec8Eeeu0xXdbba9frFj0=OqFfea0dXdd9vqai=hGuQ8kuc9pgc9s8qqaq=dirpe0xb9q8qiLsFr0=vr0=vr0dc8meaabaqaciaacaGaaeqabaWaaeGaeaaakeaat0uy0HwzTfgDPnwy3aqeh0uy0HwzTfgDPnwy3aacfaGae8hHWxeaaa@41FF@[∏_*i*, *Q*_(*t*)]

In this section we consider the segregation of one QTL and one multiallelic marker, with a recombination rate *c *between them. Let *X*_*i*,*Q*_(*t*) and *X*_*i*,*q*_(*t*) be the number of haplotypes (*i*, *Q*) and (*i*, *q) *in the population at generation *t*, respectively, and **X**(*t*) = (*X*_1,*Q*_(*t*),…, *X*_*I*,*Q*_(*t*), *X*_1,*q*_(*t*),…, *X*_*I*,*q*_(*t*)); we define also the vector of haplotype frequencies

∏(t)=X(t)2N(t)=(∏1,Q(t),…,∏I,Q(t),∏1,q(t),…,∏I,q(t))
MathType@MTEF@5@5@+=feaafiart1ev1aaatCvAUfKttLearuWrP9MDH5MBPbIqV92AaeXatLxBI9gBaebbnrfifHhDYfgasaacH8akY=wiFfYdH8Gipec8Eeeu0xXdbba9frFj0=OqFfea0dXdd9vqai=hGuQ8kuc9pgc9s8qqaq=dirpe0xb9q8qiLsFr0=vr0=vr0dc8meaabaqaciaacaGaaeqabaqabeGadaaakeaadaqeaaqaaiabcIcaOiabdsha0jabcMcaPaWcbeqab0Gaey4dIunakiabg2da9maalaaabaGaemiwaGLaeiikaGIaemiDaqNaeiykaKcabaGaeGOmaiJaemOta4KaeiikaGIaemiDaqNaeiykaKcaaiabg2da9iabcIcaOmaarababaGaeiikaGIaemiDaqNaeiykaKIaeiilaWIaeS47IWKaeiilaWYaaebeaeaacqGGOaakcqWG0baDcqGGPaqkaSqaaiabdMeajjabcYcaSiabdgfarbqab0Gaey4dIunakiabcYcaSmaarababaGaeiikaGIaemiDaqNaeiykaKcaleaacqaIXaqmcqGGSaalcqWGXbqCaeqaniabg+GivdGccqGGSaalcqWIVlctcqGGSaaldaqeqaqaaiabcIcaOiabdsha0jabcMcaPiabcMcaPaWcbaGaemysaKKaeiilaWIaemyCaehabeqdcqGHpis1aaWcbaGaeGymaeJaeiilaWIaemyuaefabeqdcqGHpis1aaaa@67E6@

These vectors are stochastic processes of time. We first present a two-locus Wright-Fisher model [46,47] that describes the distribution of **X**(*t *+ 1) given **X**(*t*). From this model and under the assumption that the allelic frequency ∏_*i*_(*t*) = ∏_*i*,*Q*_(*t*) + ∏_*i*,*q*_(*t*) is deterministic and time invariant, we deduce a recursive relation between E
 MathType@MTEF@5@5@+=feaafiart1ev1aaatCvAUfKttLearuWrP9MDH5MBPbIqV92AaeXatLxBI9gBamrtHrhAL1wy0L2yHvtyaeHbnfgDOvwBHrxAJfwnaebbnrfifHhDYfgasaacH8akY=wiFfYdH8Gipec8Eeeu0xXdbba9frFj0=OqFfea0dXdd9vqai=hGuQ8kuc9pgc9s8qqaq=dirpe0xb9q8qiLsFr0=vr0=vr0dc8meaabaqaciaacaGaaeqabaWaaeGaeaaakeaat0uy0HwzTfgDPnwy3aqeh0uy0HwzTfgDPnwy3aacfaGae8hHWxeaaa@41FF@[**∏**(*t *+ 1)] and E
 MathType@MTEF@5@5@+=feaafiart1ev1aaatCvAUfKttLearuWrP9MDH5MBPbIqV92AaeXatLxBI9gBamrtHrhAL1wy0L2yHvtyaeHbnfgDOvwBHrxAJfwnaebbnrfifHhDYfgasaacH8akY=wiFfYdH8Gipec8Eeeu0xXdbba9frFj0=OqFfea0dXdd9vqai=hGuQ8kuc9pgc9s8qqaq=dirpe0xb9q8qiLsFr0=vr0=vr0dc8meaabaqaciaacaGaaeqabaWaaeGaeaaakeaat0uy0HwzTfgDPnwy3aqeh0uy0HwzTfgDPnwy3aacfaGae8hHWxeaaa@41FF@[**∏**(*t*)] that we use to determine the expression for E
 MathType@MTEF@5@5@+=feaafiart1ev1aaatCvAUfKttLearuWrP9MDH5MBPbIqV92AaeXatLxBI9gBamrtHrhAL1wy0L2yHvtyaeHbnfgDOvwBHrxAJfwnaebbnrfifHhDYfgasaacH8akY=wiFfYdH8Gipec8Eeeu0xXdbba9frFj0=OqFfea0dXdd9vqai=hGuQ8kuc9pgc9s8qqaq=dirpe0xb9q8qiLsFr0=vr0=vr0dc8meaabaqaciaacaGaaeqabaWaaeGaeaaakeaat0uy0HwzTfgDPnwy3aqeh0uy0HwzTfgDPnwy3aacfaGae8hHWxeaaa@41FF@[∏_*i*,*Q*_(*t*)].

In the two-locus Wright-Fisher model, the effective population size *N*(*t*) is a deterministic function of time and the vector **X**(*t *+ 1) follows, conditional on **X**(*t*), a multinomial distribution with parameters (2*N*(*t *+ 1), *r*_1,*Q*_(*t*),…, *r*_*I*,*Q*_(*t*), *r*_1,*q*_(*t*),…, *r*_*I*,*q*_(*t*)), where

*r*_*i*,*Q*_(*t*) = (1 - *c*)∏_*i*,*Q*_(*t*) + *c*∏_*Q*_(*t*)∏_*i*_(*t*)

The two terms of this formula represent the probabilities of choosing nonrecombining and recombining haplotypes.

From the properties of multinomial distributions we have E
 MathType@MTEF@5@5@+=feaafiart1ev1aaatCvAUfKttLearuWrP9MDH5MBPbIqV92AaeXatLxBI9gBamrtHrhAL1wy0L2yHvtyaeHbnfgDOvwBHrxAJfwnaebbnrfifHhDYfgasaacH8akY=wiFfYdH8Gipec8Eeeu0xXdbba9frFj0=OqFfea0dXdd9vqai=hGuQ8kuc9pgc9s8qqaq=dirpe0xb9q8qiLsFr0=vr0=vr0dc8meaabaqaciaacaGaaeqabaWaaeGaeaaakeaat0uy0HwzTfgDPnwy3aqeh0uy0HwzTfgDPnwy3aacfaGae8hHWxeaaa@41FF@[*X*_*i*,*Q*_(*t *+ 1) | **X**(t)] = 2*N*(*t *+ 1)*r*_*i*,*Q*_(*t*), and thus E
 MathType@MTEF@5@5@+=feaafiart1ev1aaatCvAUfKttLearuWrP9MDH5MBPbIqV92AaeXatLxBI9gBamrtHrhAL1wy0L2yHvtyaeHbnfgDOvwBHrxAJfwnaebbnrfifHhDYfgasaacH8akY=wiFfYdH8Gipec8Eeeu0xXdbba9frFj0=OqFfea0dXdd9vqai=hGuQ8kuc9pgc9s8qqaq=dirpe0xb9q8qiLsFr0=vr0=vr0dc8meaabaqaciaacaGaaeqabaWaaeGaeaaakeaat0uy0HwzTfgDPnwy3aqeh0uy0HwzTfgDPnwy3aacfaGae8hHWxeaaa@41FF@[∏_*i*,*Q*_(*t *+ 1) | **X**(*t*)] = *r*_*i*,*Q*_(*t*). A classical result on conditional probabilities yields

E[∏i,Q(t+1)]=E[E[∏i,Q(t+1)|X(t)]]=E[ri,Q(t)]=(1−c)E[∏i,Q(t)]+cE[∏Q(t)∏i(t)]
MathType@MTEF@5@5@+=feaafiart1ev1aaatCvAUfKttLearuWrP9MDH5MBPbIqV92AaeXatLxBI9gBamrtHrhAL1wy0L2yHvtyaeHbnfgDOvwBHrxAJfwnaebbnrfifHhDYfgasaacH8akY=wiFfYdH8Gipec8Eeeu0xXdbba9frFj0=OqFfea0dXdd9vqai=hGuQ8kuc9pgc9s8qqaq=dirpe0xb9q8qiLsFr0=vr0=vr0dc8meaabaqaciaacaGaaeqabaWaaeGaeaaakeaafaqaaeWadaaabaWenfgDOvwBHrxAJf2naeXbnfgDOvwBHrxAJf2naGqbaiab=ri8fjabcUfaBnaarababaGaeiikaGIaemiDaqNaey4kaSIaeGymaeJaeiykaKIaeiyxa0faleaacqWGPbqAcqGGSaalcqWGrbquaeqaniabg+GivdaakeaacqGH9aqpaeaacqWFecFrcqGGBbWwcqWFecFrcqGGBbWwdaqeqaqaaiabcIcaOiabdsha0jabgUcaRiabigdaXiabcMcaPiabcYha8nXvP5wqSXMqHnxAJn0BKvguHDwzZbqeiyvzYrwyUfgaiyqacaGFybGaeiikaGIaemiDaqNaeiykaKIaeiyxa0Laeiyxa0faleaacqWGPbqAcqGGSaalcqWGrbquaeqaniabg+GivdaakeaaaeaacqGH9aqpaeaacqWFecFrcqGGBbWwcqWGYbGCdaWgaaWcbaGaemyAaKMaeiilaWIaemyuaefabeaakiabcIcaOiabdsha0jabcMcaPiabc2faDbqaaaqaaiabg2da9aqaaiabcIcaOiabigdaXiabgkHiTiabdogaJjabcMcaPiab=ri8fjabcUfaBnaarababaGaeiikaGIaemiDaqNaeiykaKIaeiyxa0Laey4kaSIaem4yamMae8hHWxKaei4waS1aaebeaeaacqGGOaakcqWG0baDcqGGPaqkdaqeqaqaaiabcIcaOiabdsha0jabcMcaPiabc2faDbWcbaGaemyAaKgabeqdcqGHpis1aaWcbaGaemyuaefabeqdcqGHpis1aaWcbaGaemyAaKMaeiilaWIaemyuaefabeqdcqGHpis1aaaaaaa@A7DC@

We assume that ∏_*i*_(*t*) = ∏_*i *_is time invariant, which is reasonable because allele *i *is supposed to be much older than allele *Q *and consequently its frequency is much higher. This leads to

E
 MathType@MTEF@5@5@+=feaafiart1ev1aaatCvAUfKttLearuWrP9MDH5MBPbIqV92AaeXatLxBI9gBamrtHrhAL1wy0L2yHvtyaeHbnfgDOvwBHrxAJfwnaebbnrfifHhDYfgasaacH8akY=wiFfYdH8Gipec8Eeeu0xXdbba9frFj0=OqFfea0dXdd9vqai=hGuQ8kuc9pgc9s8qqaq=dirpe0xb9q8qiLsFr0=vr0=vr0dc8meaabaqaciaacaGaaeqabaWaaeGaeaaakeaat0uy0HwzTfgDPnwy3aqeh0uy0HwzTfgDPnwy3aacfaGae8hHWxeaaa@41FF@[∏_*i*,*Q*_(*t *+ 1)] = (1 - *c*)E
 MathType@MTEF@5@5@+=feaafiart1ev1aaatCvAUfKttLearuWrP9MDH5MBPbIqV92AaeXatLxBI9gBamrtHrhAL1wy0L2yHvtyaeHbnfgDOvwBHrxAJfwnaebbnrfifHhDYfgasaacH8akY=wiFfYdH8Gipec8Eeeu0xXdbba9frFj0=OqFfea0dXdd9vqai=hGuQ8kuc9pgc9s8qqaq=dirpe0xb9q8qiLsFr0=vr0=vr0dc8meaabaqaciaacaGaaeqabaWaaeGaeaaakeaat0uy0HwzTfgDPnwy3aqeh0uy0HwzTfgDPnwy3aacfaGae8hHWxeaaa@41FF@[∏_*i*,*Q*_(*t*)] + *c*E
 MathType@MTEF@5@5@+=feaafiart1ev1aaatCvAUfKttLearuWrP9MDH5MBPbIqV92AaeXatLxBI9gBamrtHrhAL1wy0L2yHvtyaeHbnfgDOvwBHrxAJfwnaebbnrfifHhDYfgasaacH8akY=wiFfYdH8Gipec8Eeeu0xXdbba9frFj0=OqFfea0dXdd9vqai=hGuQ8kuc9pgc9s8qqaq=dirpe0xb9q8qiLsFr0=vr0=vr0dc8meaabaqaciaacaGaaeqabaWaaeGaeaaakeaat0uy0HwzTfgDPnwy3aqeh0uy0HwzTfgDPnwy3aacfaGae8hHWxeaaa@41FF@[∏_*Q*_(*t*)]∏_*i*_

and the entire vector **∏**(*t*) satisfies

E
 MathType@MTEF@5@5@+=feaafiart1ev1aaatCvAUfKttLearuWrP9MDH5MBPbIqV92AaeXatLxBI9gBamrtHrhAL1wy0L2yHvtyaeHbnfgDOvwBHrxAJfwnaebbnrfifHhDYfgasaacH8akY=wiFfYdH8Gipec8Eeeu0xXdbba9frFj0=OqFfea0dXdd9vqai=hGuQ8kuc9pgc9s8qqaq=dirpe0xb9q8qiLsFr0=vr0=vr0dc8meaabaqaciaacaGaaeqabaWaaeGaeaaakeaat0uy0HwzTfgDPnwy3aqeh0uy0HwzTfgDPnwy3aacfaGae8hHWxeaaa@41FF@[**∏**(*t *+ 1)] = E
 MathType@MTEF@5@5@+=feaafiart1ev1aaatCvAUfKttLearuWrP9MDH5MBPbIqV92AaeXatLxBI9gBamrtHrhAL1wy0L2yHvtyaeHbnfgDOvwBHrxAJfwnaebbnrfifHhDYfgasaacH8akY=wiFfYdH8Gipec8Eeeu0xXdbba9frFj0=OqFfea0dXdd9vqai=hGuQ8kuc9pgc9s8qqaq=dirpe0xb9q8qiLsFr0=vr0=vr0dc8meaabaqaciaacaGaaeqabaWaaeGaeaaakeaat0uy0HwzTfgDPnwy3aqeh0uy0HwzTfgDPnwy3aacfaGae8hHWxeaaa@41FF@[**∏**(*t*)] (*cA *+ (1 - *c*)*Id*_*I*_)     (6)

where *A *= (∏_1_,…, ∏_*I*_) l
 MathType@MTEF@5@5@+=feaafiart1ev1aaatCvAUfKttLearuWrP9MDH5MBPbIqV92AaeXatLxBI9gBamrtHrhAL1wy0L2yHvtyaeHbnfgDOvwBHrxAJfwnaebbnrfifHhDYfgasaacH8akY=wiFfYdH8Gipec8Eeeu0xXdbba9frFj0=OqFfea0dXdd9vqai=hGuQ8kuc9pgc9s8qqaq=dirpe0xb9q8qiLsFr0=vr0=vr0dc8meaabaqaciaacaGaaeqabaWaaeGaeaaakeaat0uy0HwzTfgDPnwy3aqeh0uy0HwzTfgDPnwy3aacfaGae8xPWZgaaa@4247@_*I*_, where  is the Kronecker product, l
 MathType@MTEF@5@5@+=feaafiart1ev1aaatCvAUfKttLearuWrP9MDH5MBPbIqV92AaeXatLxBI9gBamrtHrhAL1wy0L2yHvtyaeHbnfgDOvwBHrxAJfwnaebbnrfifHhDYfgasaacH8akY=wiFfYdH8Gipec8Eeeu0xXdbba9frFj0=OqFfea0dXdd9vqai=hGuQ8kuc9pgc9s8qqaq=dirpe0xb9q8qiLsFr0=vr0=vr0dc8meaabaqaciaacaGaaeqabaWaaeGaeaaakeaat0uy0HwzTfgDPnwy3aqeh0uy0HwzTfgDPnwy3aacfaGae8xPWZgaaa@4247@_*I *_is the column vector of size *I *with all components equal to 1, and *Id*_*I *_is the identity matrix of size *I *× *I*.

*A *is idempotent since ∑i=1I∏i=1
MathType@MTEF@5@5@+=feaafiart1ev1aaatCvAUfKttLearuWrP9MDH5MBPbIqV92AaeXatLxBI9gBaebbnrfifHhDYfgasaacH8akY=wiFfYdH8Gipec8Eeeu0xXdbba9frFj0=OqFfea0dXdd9vqai=hGuQ8kuc9pgc9s8qqaq=dirpe0xb9q8qiLsFr0=vr0=vr0dc8meaabaqaciaacaGaaeqabaqabeGadaaakeaadaaeWaqaamaarababaGaeyypa0JaeGymaedaleaacqWGPbqAaeqaniabg+GivdaaleaacqWGPbqAcqGH9aqpcqaIXaqmaeaacqWGjbqsa0GaeyyeIuoaaaa@383D@, and so we can prove by recurrence on *t *that

E
 MathType@MTEF@5@5@+=feaafiart1ev1aaatCvAUfKttLearuWrP9MDH5MBPbIqV92AaeXatLxBI9gBamrtHrhAL1wy0L2yHvtyaeHbnfgDOvwBHrxAJfwnaebbnrfifHhDYfgasaacH8akY=wiFfYdH8Gipec8Eeeu0xXdbba9frFj0=OqFfea0dXdd9vqai=hGuQ8kuc9pgc9s8qqaq=dirpe0xb9q8qiLsFr0=vr0=vr0dc8meaabaqaciaacaGaaeqabaWaaeGaeaaakeaat0uy0HwzTfgDPnwy3aqeh0uy0HwzTfgDPnwy3aacfaGae8hHWxeaaa@41FF@[**∏**(*t*)] = E
 MathType@MTEF@5@5@+=feaafiart1ev1aaatCvAUfKttLearuWrP9MDH5MBPbIqV92AaeXatLxBI9gBamrtHrhAL1wy0L2yHvtyaeHbnfgDOvwBHrxAJfwnaebbnrfifHhDYfgasaacH8akY=wiFfYdH8Gipec8Eeeu0xXdbba9frFj0=OqFfea0dXdd9vqai=hGuQ8kuc9pgc9s8qqaq=dirpe0xb9q8qiLsFr0=vr0=vr0dc8meaabaqaciaacaGaaeqabaWaaeGaeaaakeaat0uy0HwzTfgDPnwy3aqeh0uy0HwzTfgDPnwy3aacfaGae8hHWxeaaa@41FF@[**∏**(0)] ((1 - (1 - *c*)^*t*^) *A *+ (1 - *c*)^*t *^*Id*_*I*_)

Taking the *i*th coordinate we get

E
 MathType@MTEF@5@5@+=feaafiart1ev1aaatCvAUfKttLearuWrP9MDH5MBPbIqV92AaeXatLxBI9gBamrtHrhAL1wy0L2yHvtyaeHbnfgDOvwBHrxAJfwnaebbnrfifHhDYfgasaacH8akY=wiFfYdH8Gipec8Eeeu0xXdbba9frFj0=OqFfea0dXdd9vqai=hGuQ8kuc9pgc9s8qqaq=dirpe0xb9q8qiLsFr0=vr0=vr0dc8meaabaqaciaacaGaaeqabaWaaeGaeaaakeaat0uy0HwzTfgDPnwy3aqeh0uy0HwzTfgDPnwy3aacfaGae8hHWxeaaa@41FF@[**∏**_*i*,*Q*_(*t*)] = (1 - *c*)^*t*^∏_*i*,*Q*_(0) + (1 - (1 - *c*)^*t*^) ∏_*Q*_(0)∏_*i *_    (7)

### Derivation of the formula for E[∏i1,Q,i2(t)]
MathType@MTEF@5@5@+=feaafiart1ev1aaatCvAUfKttLearuWrP9MDH5MBPbIqV92AaeXatLxBI9gBamrtHrhAL1wy0L2yHvtyaeHbnfgDOvwBHrxAJfwnaebbnrfifHhDYfgasaacH8akY=wiFfYdH8Gipec8Eeeu0xXdbba9frFj0=OqFfea0dXdd9vqai=hGuQ8kuc9pgc9s8qqaq=dirpe0xb9q8qiLsFr0=vr0=vr0dc8meaabaqaciaacaGaaeqabaWaaeGaeaaakeaat0uy0HwzTfgDPnwy3aqeh0uy0HwzTfgDPnwy3aacfaGae8hHWxKaei4waS1aaebeaeaacqGGOaakcqWG0baDcqGGPaqkaSqaaiabdMgaPnaaBaaameaacqaIXaqmaeqaaSGaeiilaWIaemyuaeLaeiilaWIaemyAaK2aaSbaaWqaaiabikdaYaqabaaaleqaniabg+GivdGccqGGDbqxaaa@5171@

We now consider the more complex case of two multiallelic markers flanking the QTL. We proceed as in the previous section, defining first a three-locus Wright-Fisher model and then deducing from it a recurrence relation for the expected value of haplotype frequencies. To do this we also assume that the markers are in equilibrium. From the recurrence relation we finally obtain the expression for E[∏i1,Q,i2(t)]
MathType@MTEF@5@5@+=feaafiart1ev1aaatCvAUfKttLearuWrP9MDH5MBPbIqV92AaeXatLxBI9gBamrtHrhAL1wy0L2yHvtyaeHbnfgDOvwBHrxAJfwnaebbnrfifHhDYfgasaacH8akY=wiFfYdH8Gipec8Eeeu0xXdbba9frFj0=OqFfea0dXdd9vqai=hGuQ8kuc9pgc9s8qqaq=dirpe0xb9q8qiLsFr0=vr0=vr0dc8meaabaqaciaacaGaaeqabaWaaeGaeaaakeaat0uy0HwzTfgDPnwy3aqeh0uy0HwzTfgDPnwy3aacfaGae8hHWxKaei4waS1aaebeaeaacqGGOaakcqWG0baDcqGGPaqkaSqaaiabdMgaPnaaBaaameaacqaIXaqmaeqaaSGaeiilaWIaemyuaeLaeiilaWIaemyAaK2aaSbaaWqaaiabikdaYaqabaaaleqaniabg+GivdGccqGGDbqxaaa@5171@.

The three-locus Wright-Fisher model describes the segregation of haplotypes composed of the QTL and two flanking markers. The first marker has *I*_1 _alleles and a recombination rate *c*_1 _with the QTL; the second one has *I*_2 _alleles and a recombination rate *c*_2 _with the QTL. We denote Xi1,Q,i2(t)
 MathType@MTEF@5@5@+=feaafiart1ev1aaatCvAUfKttLearuWrP9MDH5MBPbIqV92AaeXatLxBI9gBaebbnrfifHhDYfgasaacH8akY=wiFfYdH8Gipec8Eeeu0xXdbba9frFj0=OqFfea0dXdd9vqai=hGuQ8kuc9pgc9s8qqaq=dirpe0xb9q8qiLsFr0=vr0=vr0dc8meaabaqaciaacaGaaeqabaqabeGadaaakeaacqWGybawdaWgaaWcbaGaemyAaK2aaSbaaWqaaiabigdaXaqabaWccqGGSaalcqWGrbqucqGGSaalcqWGPbqAdaWgaaadbaGaeGOmaidabeaaaSqabaGccqGGOaakcqWG0baDcqGGPaqkaaa@3931@, *i*_1 _= 1,…, *I*_1_, *i*_2 _= 1,…, *I*_2_, as the number of copies of haplotype (*i*_1_, *Q*, *i*_2_) in the population at generation *t*, and ∏i1,Q,i2(t)
MathType@MTEF@5@5@+=feaafiart1ev1aaatCvAUfKttLearuWrP9MDH5MBPbIqV92AaeXatLxBI9gBaebbnrfifHhDYfgasaacH8akY=wiFfYdH8Gipec8Eeeu0xXdbba9frFj0=OqFfea0dXdd9vqai=hGuQ8kuc9pgc9s8qqaq=dirpe0xb9q8qiLsFr0=vr0=vr0dc8meaabaqaciaacaGaaeqabaqabeGadaaakeaadaqeqaqaaiabcIcaOiabdsha0jabcMcaPaWcbaGaemyAaK2aaSbaaWqaaiabigdaXaqabaWccqGGSaalcqWGrbqucqGGSaalcqWGPbqAdaWgaaadbaGaeGOmaidabeaaaSqab0Gaey4dIunaaaa@3994@ as the corresponding frequency. **X**(*t *+ 1) has dimension 2*I*_1_*I*_2_, but still has a multinomial distribution given **X**(*t*) with parameters (2N(t+1),r1,Q,1(t),…,rI1,Q,I2(t),r1,q,1(t),…,rI1,q,I2(t))
 MathType@MTEF@5@5@+=feaafiart1ev1aaatCvAUfKttLearuWrP9MDH5MBPbIqV92AaeXatLxBI9gBaebbnrfifHhDYfgasaacH8akY=wiFfYdH8Gipec8Eeeu0xXdbba9frFj0=OqFfea0dXdd9vqai=hGuQ8kuc9pgc9s8qqaq=dirpe0xb9q8qiLsFr0=vr0=vr0dc8meaabaqaciaacaGaaeqabaqabeGadaaakeaacqGGOaakcqaIYaGmcqWGobGtcqGGOaakcqWG0baDcqGHRaWkcqaIXaqmcqGGPaqkcqGGSaalcqWGYbGCdaWgaaWcbaGaeGymaeJaeiilaWIaemyuaeLaeiilaWIaeGymaedabeaakiabcIcaOiabdsha0jabcMcaPiabcYcaSiabl+UimjabcYcaSiabdkhaYnaaBaaaleaacqWGjbqsdaWgaaadbaGaeGymaedabeaaliabcYcaSiabdgfarjabcYcaSiabdMeajnaaBaaameaacqaIYaGmaeqaaaWcbeaakiabcIcaOiabdsha0jabcMcaPiabcYcaSiabdkhaYnaaBaaaleaacqaIXaqmcqGGSaalcqWGXbqCcqGGSaalcqaIXaqmaeqaaOGaeiikaGIaemiDaqNaeiykaKIaeiilaWIaeS47IWKaeiilaWIaemOCai3aaSbaaSqaaiabdMeajnaaBaaameaacqaIXaqmcqGGSaalcqWGXbqCcqGGSaalaeqaaSGaemysaK0aaSbaaWqaaiabikdaYaqabaaaleqaaOGaeiikaGIaemiDaqNaeiykaKIaeiykaKcaaa@6A9A@, where ri1,Q,i2(t)=(1−c1)(1−c2)∏i1,Q,i2(t)+c1(1−c2)∏i1∏Q,i2(t)+c2(1−c1)∏i2∏i1,Q(t)+c1c2∏i1,i2(t)∏Q(t)
MathType@MTEF@5@5@+=feaafiart1ev1aaatCvAUfKttLearuWrP9MDH5MBPbIqV92AaeXatLxBI9gBaebbnrfifHhDYfgasaacH8akY=wiFfYdH8Gipec8Eeeu0xXdbba9frFj0=OqFfea0dXdd9vqai=hGuQ8kuc9pgc9s8qqaq=dirpe0xb9q8qiLsFr0=vr0=vr0dc8meaabaqaciaacaGaaeqabaqabeGadaaakeaacqWGYbGCdaWgaaWcbaGaemyAaK2aaSbaaWqaaiabigdaXaqabaWccqGGSaalcqWGrbqucqGGSaalcqWGPbqAdaWgaaadbaGaeGOmaidabeaaaSqabaGccqGGOaakcqWG0baDcqGGPaqkcqGH9aqpcqGGOaakcqaIXaqmcqGHsislcqWGJbWydaWgaaWcbaGaeGymaedabeaakiabcMcaPiabcIcaOiabigdaXiabgkHiTiabdogaJnaaBaaaleaacqaIYaGmaeqaaOGaeiykaKYaaebeaeaacqGGOaakcqWG0baDcqGGPaqkcqGHRaWkcqWGJbWydaWgaaWcbaGaeGymaedabeaaaeaacqWGPbqAdaWgaaadbaGaeGymaedabeaaliabcYcaSiabdgfarjabcYcaSiabdMgaPnaaBaaameaacqaIYaGmaeqaaaWcbeqdcqGHpis1aOGaeiikaGIaeGymaeJaeyOeI0Iaem4yam2aaSbaaSqaaiabikdaYaqabaGccqGGPaqkdaqeqaqaamaarababaGaeiikaGIaemiDaqNaeiykaKIaey4kaSIaem4yam2aaSbaaSqaaiabikdaYaqabaaabaGaemyuaeLaeiilaWIaemyAaK2aaSbaaWqaaiabikdaYaqabaaaleqaniabg+GivdaaleaacqWGPbqAdaWgaaadbaGaeGymaedabeaaaSqab0Gaey4dIunakiabcIcaOiabigdaXiabgkHiTiabdogaJnaaBaaaleaacqaIXaqmaeqaaOGaeiykaKYaaebeaeaadaqeqaqaaiabcIcaOiabdsha0jabcMcaPaWcbaGaemyAaK2aaSbaaWqaaiabigdaXaqabaWccqGGSaalcqWGrbquaeqaniabg+GivdaaleaacqWGPbqAdaWgaaadbaGaeGOmaidabeaaaSqab0Gaey4dIunakiabgUcaRiabdogaJnaaBaaaleaacqaIXaqmaeqaaOGaem4yam2aaSbaaSqaaiabikdaYaqabaGcdaqeqaqaaiabcIcaOiabdsha0jabcMcaPmaarababaGaeiikaGIaemiDaqNaeiykaKcaleaacqWGrbquaeqaniabg+GivdaaleaacqWGPbqAdaWgaaadbaGaeGymaedabeaaliabcYcaSiabdMgaPnaaBaaameaacqaIYaGmaeqaaaWcbeqdcqGHpis1aaaa@988A@, ∏i1
MathType@MTEF@5@5@+=feaafiart1ev1aaatCvAUfeBSjuyZL2yd9gzLbvyNv2Caerbhv2BYDwAHbqedmvETj2BSbqee0evGueE0jxyaibaiKI8=vI8tuQ8FMI8Gi=hEeeu0xXdbba9frFj0=OqFfea0dXdd9vqai=hGuQ8kuc9pgc9s8qqaq=dirpe0xb9q8qiLsFr0=vr0=vr0dc8meaabaqaciGacaGaaeqabaqadeqadaaakeaadaqeqaqaaaWcbaGaamyAamaaBaaameaacaaIXaaabeaaaSqab0Gaey4dIunaaaa@36E0@, ∏i2
MathType@MTEF@5@5@+=feaafiart1ev1aaatCvAUfeBSjuyZL2yd9gzLbvyNv2CaerbwvMCKfMBHbqedmvETj2BSbqee0evGueE0jxyaibaieYdOi=BH8vipeYdI8qiW7rqqrFfpeea0xe9Lq=Jc9vqaqpepm0xbbG8FasPYRqj0=yi0lXdbba9pGe9qqFf0dXdHuk9fr=xfr=xfrpiWZqaaeaabiGaaiaacaqabeaabeqacmaaaOqaamaarababaaaleaacaWGPbWaaSbaaWqaaiaaikdaaeqaaaWcbeqdcqGHpis1aaaa@3376@, and ∏_*Q*_(*t*) are the marginal frequencies of alleles *i*_1 _at the left marker, *i*_2 _at the right marker, and *Q *at the QTL, respectively, and ∏i1,Q(t)
MathType@MTEF@5@5@+=feaafiart1ev1aaatCvAUfKttLearuWrP9MDH5MBPbIqV92AaeXatLxBI9gBaebbnrfifHhDYfgasaacH8akY=wiFfYdH8Gipec8Eeeu0xXdbba9frFj0=OqFfea0dXdd9vqai=hGuQ8kuc9pgc9s8qqaq=dirpe0xb9q8qiLsFr0=vr0=vr0dc8meaabaqaciaacaGaaeqabaqabeGadaaakeaadaqeqaqaaiabcIcaOiabdsha0jabcMcaPaWcbaGaemyAaK2aaSbaaWqaaiabigdaXaqabaWccqGGSaalcqWGrbquaeqaniabg+Givdaaaa@362F@ and ∏Q,i2(t)
MathType@MTEF@5@5@+=feaafiart1ev1aaatCvAUfKttLearuWrP9MDH5MBPbIqV92AaeXatLxBI9gBaebbnrfifHhDYfgasaacH8akY=wiFfYdH8Gipec8Eeeu0xXdbba9frFj0=OqFfea0dXdd9vqai=hGuQ8kuc9pgc9s8qqaq=dirpe0xb9q8qiLsFr0=vr0=vr0dc8meaabaqaciaacaGaaeqabaqabeGadaaakeaadaqeqaqaaiabcIcaOiabdsha0jabcMcaPaWcbaGaemyuaeLaeiilaWIaemyAaK2aaSbaaWqaaiabikdaYaqabaaaleqaniabg+Givdaaaa@3631@ are the marginal frequencies of haplotypes (*i*_1_, *Q*) and (*Q*, *i*_2_), respectively. The four terms in this formula correspond to the different origins of haplotypes (*i*_1 _*Q*, *i*_2_) at generation *t *+ 1: nonrecombining, recombining between QTL and the left-side marker, recombining between QTL and the right-side marker, and double recombining.

As in the previous section, we can express the expected value of the frequencies of haplotypes at time *t *+ 1 as

E[∏i1,Q,i2(t+1)]=E[E[∏i1,Q,i2(t+1)|X(t)]]=E[ri1,Q,i2(t)]=(1−c1)(1−c2)E[∏i1,Q,i2(t)]+c1(1−c2)∏i1E[∏Q,i2(t)]+c2(1−c1)∏i2E[∏i1,Q(t)]+c1c2E[∏i1,i2(t)∏Q(t)]
MathType@MTEF@5@5@+=feaafiart1ev1aaatCvAUfKttLearuWrP9MDH5MBPbIqV92AaeXatLxBI9gBamrtHrhAL1wy0L2yHvtyaeHbnfgDOvwBHrxAJfwnaebbnrfifHhDYfgasaacH8akY=wiFfYdH8Gipec8Eeeu0xXdbba9frFj0=OqFfea0dXdd9vqai=hGuQ8kuc9pgc9s8qqaq=dirpe0xb9q8qiLsFr0=vr0=vr0dc8meaabaqaciaacaGaaeqabaWaaeGaeaaakeaafaqaaeabdaaaaeaat0uy0HwzTfgDPnwy3aqeh0uy0HwzTfgDPnwy3aacfaGae8hHWxKaei4waS1aaebeaeaacqGGOaakcqWG0baDcqGHRaWkcqaIXaqmcqGGPaqkcqGGDbqxaSqaaiabdMgaPnaaBaaameaacqaIXaqmaeqaaSGaeiilaWIaemyuaeLaeiilaWIaemyAaK2aaSbaaWqaaiabikdaYaqabaaaleqaniabg+GivdaakeaacqGH9aqpaeaafaqabeqadaaabaGae8hHWxKaei4waSLae8hHWxKaei4waS1aaebeaeaacqGGOaakcqWG0baDcqGHRaWkcqaIXaqmcqGGPaqkcqGG8baFcqWGybawcqGGOaakcqWG0baDcqGGPaqkcqGGDbqxcqGGDbqxaSqaaiabdMgaPnaaBaaameaacqaIXaqmaeqaaSGaeiilaWIaemyuaeLaeiilaWIaemyAaK2aaSbaaWqaaiabikdaYaqabaaaleqaniabg+GivdaakeaaaeaaaaaabaaabaGaeyypa0dabaGae8hHWxKaei4waSLaemOCai3aaSbaaSqaaiabdMgaPnaaBaaameaacqaIXaqmaeqaaSGaeiilaWIaemyuaeLaeiilaWIaemyAaK2aaSbaaWqaaiabikdaYaqabaaaleqaaOGaeiikaGIaemiDaqNaeiykaKIaeiyxa0fabaaabaGaeyypa0dabaGaeiikaGIaeGymaeJaeyOeI0Iaem4yam2aaSbaaSqaaiabigdaXaqabaGccqGGPaqkcqGGOaakcqaIXaqmcqGHsislcqWGJbWydaWgaaWcbaGaeGOmaidabeaakiabcMcaPiab=ri8fjabcUfaBnaarababaGaeiikaGIaemiDaqNaeiykaKcaleaacqWGPbqAdaWgaaadbaGaeGymaedabeaaliabcYcaSiabdgfarjabcYcaSiabdMgaPnaaBaaameaacqaIYaGmaeqaaaWcbeqdcqGHpis1aOGaeiyxa0Laey4kaSIaem4yam2aaSbaaSqaaiabigdaXaqabaGccqGGOaakcqaIXaqmcqGHsislcqWGJbWydaWgaaWcbaGaeGOmaidabeaakiabcMcaPmaarababaGae8hHWxKaei4waS1aaebeaeaacqGGOaakcqWG0baDcqGGPaqkcqGGDbqxaSqaaiabdgfarjabcYcaSiabdMgaPnaaBaaameaacqaIYaGmaeqaaaWcbeqdcqGHpis1aaWcbaGaemyAaK2aaSbaaWqaaiabigdaXaqabaaaleqaniabg+GivdaakeaaaeaacqGHRaWkaeaacqWGJbWydaWgaaWcbaGaeGOmaidabeaakiabcIcaOiabigdaXiabgkHiTiabdogaJnaaBaaaleaacqaIXaqmaeqaaOGaeiykaKYaaebeaeaacqWFecFrcqGGBbWwdaqeqaqaaiabcIcaOiabdsha0jabcMcaPiabc2faDjabgUcaRiabdogaJnaaBaaaleaacqaIXaqmaeqaaOGaem4yam2aaSbaaSqaaiabikdaYaqabaGccqWFecFrcqGGBbWwdaqeqaqaaiabcIcaOiabdsha0jabcMcaPmaarababaGaeiikaGIaemiDaqNaeiykaKcaleaacqWGrbquaeqaniabg+GivdGccqGGDbqxaSqaaiabdMgaPnaaBaaameaacqaIXaqmaeqaaSGaeiilaWIaemyAaK2aaSbaaWqaaiabikdaYaqabaaaleqaniabg+GivdaaleaacqWGPbqAdaWgaaadbaGaeGymaedabeaaliabcYcaSiabdgfarbqab0Gaey4dIunaaSqaaiabdMgaPnaaBaaameaacqaIYaGmaeqaaaWcbeqdcqGHpis1aaaaaaa@F752@

Assuming that the markers are in equilibrium and that the allelic frequencies are constant; i.e.,

∏i1,i2(t)=∏i1(t)∏i2(t)=∏i1∏i2
MathType@MTEF@5@5@+=feaafiart1ev1aaatCvAUfKttLearuWrP9MDH5MBPbIqV92AaeXatLxBI9gBaebbnrfifHhDYfgasaacH8akY=wiFfYdH8Gipec8Eeeu0xXdbba9frFj0=OqFfea0dXdd9vqai=hGuQ8kuc9pgc9s8qqaq=dirpe0xb9q8qiLsFr0=vr0=vr0dc8meaabaqaciaacaGaaeqabaqabeGadaaakeaadaqeqaqaaiabcIcaOiabdsha0jabcMcaPaWcbaGaemyAaK2aaSbaaWqaaiabigdaXaqabaWccqGGSaalcqWGPbqAdaWgaaadbaGaeGOmaidabeaaaSqab0Gaey4dIunakiabg2da9maarababaGaeiikaGIaemiDaqNaeiykaKcaleaacqWGPbqAdaWgaaadbaGaeGymaedabeaaaSqab0Gaey4dIunakmaarababaGaeiikaGIaemiDaqNaeiykaKcaleaacqWGPbqAdaWgaaadbaGaeGOmaidabeaaaSqab0Gaey4dIunakiabg2da9maarababaWaaebeaeaaaSqaaiabdMgaPnaaBaaameaacqaIYaGmaeqaaaWcbeqdcqGHpis1aaWcbaGaemyAaK2aaSbaaWqaaiabigdaXaqabaaaleqaniabg+Givdaaaa@5151@

we get

E[∏i1,Q,i2(t+1)=(1−c1)(1−c2)E[∏i1,Q,i2(t)]+c1(1−c2)E[∏Q,i2(t)]∏i1+c2(1−c1)E[∏i1,Q(t)]∏i2+ c1c2E[∏Q(t)]∏i1∏i2
MathType@MTEF@5@5@+=feaafiart1ev1aaatCvAUfKttLearuWrP9MDH5MBPbIqV92AaeXatLxBI9gBamrtHrhAL1wy0L2yHvtyaeHbnfgDOvwBHrxAJfwnaebbnrfifHhDYfgasaacH8akY=wiFfYdH8Gipec8Eeeu0xXdbba9frFj0=OqFfea0dXdd9vqai=hGuQ8kuc9pgc9s8qqaq=dirpe0xb9q8qiLsFr0=vr0=vr0dc8meaabaqaciaacaGaaeqabaWaaeGaeaaakeaafaqaaeGadaaabaWenfgDOvwBHrxAJf2naeXbnfgDOvwBHrxAJf2naGqbaiab=ri8fjabcUfaBnaarababaGaeiikaGIaemiDaqNaey4kaSIaeGymaeJaeiykaKcaleaacqWGPbqAdaWgaaadbaGaeGymaedabeaaliabcYcaSiabdgfarjabcYcaSiabdMgaPnaaBaaameaacqaIYaGmaeqaaaWcbeqdcqGHpis1aaGcbaGaeyypa0dabaGaeiikaGIaeGymaeJaeyOeI0Iaem4yam2aaSbaaSqaaiabigdaXaqabaGccqGGPaqkcqGGOaakcqaIXaqmcqGHsislcqWGJbWydaWgaaWcbaGaeGOmaidabeaakiabcMcaPiab=ri8fjabcUfaBnaarababaGaeiikaGIaemiDaqNaeiykaKIaeiyxa0Laey4kaSIaem4yam2aaSbaaSqaaiabigdaXaqabaGccqGGOaakcqaIXaqmcqGHsislcqWGJbWydaWgaaWcbaGaeGOmaidabeaakiabcMcaPiab=ri8fjabcUfaBnaarababaGaeiikaGIaemiDaqNaeiykaKIaeiyxa01aaebeaeaaaSqaaiabdMgaPnaaBaaameaacqaIXaqmaeqaaaWcbeqdcqGHpis1aaWcbaGaemyuaeLaeiilaWIaemyAaK2aaSbaaWqaaiabikdaYaqabaaaleqaniabg+GivdaaleaacqWGPbqAdaWgaaadbaGaeGymaedabeaaliabcYcaSiabdgfarjabcYcaSiabdMgaPnaaBaaameaacqaIYaGmaeqaaaWcbeqdcqGHpis1aaGcbaaabaGaey4kaScabaGaem4yam2aaSbaaSqaaiabikdaYaqabaGccqGGOaakcqaIXaqmcqGHsislcqWGJbWydaWgaaWcbaGaeGymaedabeaakiabcMcaPiab=ri8fjabcUfaBnaarababaGaeiikaGIaemiDaqNaeiykaKIaeiyxa01aaebeaeaacqGHRaWkcqqGGaaicqWGJbWydaWgaaWcbaGaeGymaedabeaakiabdogaJnaaBaaaleaacqaIYaGmaeqaaOGae8hHWxKaei4waS1aaebeaeaacqGGOaakcqWG0baDcqGGPaqkcqGGDbqxdaqeqaqaamaarababaaaleaacqWGPbqAdaWgaaadbaGaeGOmaidabeaaaSqab0Gaey4dIunaaSqaaiabdMgaPnaaBaaameaacqaIXaqmaeqaaaWcbeqdcqGHpis1aaWcbaGaemyuaefabeqdcqGHpis1aaWcbaGaemyAaK2aaSbaaWqaaiabikdaYaqabaaaleqaniabg+GivdaaleaacqWGPbqAdaWgaaadbaGaeGymaedabeaaliabcYcaSiabdgfarbqab0Gaey4dIunaaaaaaa@C2DC@

Substituting E
 MathType@MTEF@5@5@+=feaafiart1ev1aaatCvAUfKttLearuWrP9MDH5MBPbIqV92AaeXatLxBI9gBamrtHrhAL1wy0L2yHvtyaeHbnfgDOvwBHrxAJfwnaebbnrfifHhDYfgasaacH8akY=wiFfYdH8Gipec8Eeeu0xXdbba9frFj0=OqFfea0dXdd9vqai=hGuQ8kuc9pgc9s8qqaq=dirpe0xb9q8qiLsFr0=vr0=vr0dc8meaabaqaciaacaGaaeqabaWaaeGaeaaakeaat0uy0HwzTfgDPnwy3aqeh0uy0HwzTfgDPnwy3aacfaGae8hHWxeaaa@41FF@[∏i1,Q(t)
MathType@MTEF@5@5@+=feaafiart1ev1aaatCvAUfKttLearuWrP9MDH5MBPbIqV92AaeXatLxBI9gBaebbnrfifHhDYfgasaacH8akY=wiFfYdH8Gipec8Eeeu0xXdbba9frFj0=OqFfea0dXdd9vqai=hGuQ8kuc9pgc9s8qqaq=dirpe0xb9q8qiLsFr0=vr0=vr0dc8meaabaqaciaacaGaaeqabaqabeGadaaakeaadaqeqaqaaiabcIcaOiabdsha0jabcMcaPaWcbaGaemyAaK2aaSbaaWqaaiabigdaXaqabaWccqGGSaalcqWGrbquaeqaniabg+Givdaaaa@362F@] and E
 MathType@MTEF@5@5@+=feaafiart1ev1aaatCvAUfKttLearuWrP9MDH5MBPbIqV92AaeXatLxBI9gBamrtHrhAL1wy0L2yHvtyaeHbnfgDOvwBHrxAJfwnaebbnrfifHhDYfgasaacH8akY=wiFfYdH8Gipec8Eeeu0xXdbba9frFj0=OqFfea0dXdd9vqai=hGuQ8kuc9pgc9s8qqaq=dirpe0xb9q8qiLsFr0=vr0=vr0dc8meaabaqaciaacaGaaeqabaWaaeGaeaaakeaat0uy0HwzTfgDPnwy3aqeh0uy0HwzTfgDPnwy3aacfaGae8hHWxeaaa@41FF@[∏Q,i2(t)
MathType@MTEF@5@5@+=feaafiart1ev1aaatCvAUfKttLearuWrP9MDH5MBPbIqV92AaeXatLxBI9gBaebbnrfifHhDYfgasaacH8akY=wiFfYdH8Gipec8Eeeu0xXdbba9frFj0=OqFfea0dXdd9vqai=hGuQ8kuc9pgc9s8qqaq=dirpe0xb9q8qiLsFr0=vr0=vr0dc8meaabaqaciaacaGaaeqabaqabeGadaaakeaadaqeqaqaaiabcIcaOiabdsha0jabcMcaPaWcbaGaemyuaeLaeiilaWIaemyAaK2aaSbaaWqaaiabikdaYaqabaaaleqaniabg+Givdaaaa@3631@] with the expressions determined in the previous section gives

E[∏i1,Q,i2(t+1)]=(1−c1)(1−c2)E[∏i1,Q,i2(t)]+β2c1(1−c2)t+1+β1c2(1−c1)t+1+(c1(1−c2)+c2(1−c1)+c1c2)∏Q(0)∏i1∏i2     (8)
MathType@MTEF@5@5@+=feaafiart1ev1aaatCvAUfKttLearuWrP9MDH5MBPbIqV92AaeXatLxBI9gBamrtHrhAL1wy0L2yHvtyaeHbnfgDOvwBHrxAJfwnaebbnrfifHhDYfgasaacH8akY=wiFfYdH8Gipec8Eeeu0xXdbba9frFj0=OqFfea0dXdd9vqai=hGuQ8kuc9pgc9s8qqaq=dirpe0xb9q8qiLsFr0=vr0=vr0dc8meaabaqaciaacaGaaeqabaWaaeGaeaaakeaafaqaaeGadaaabaWenfgDOvwBHrxAJf2naeXbnfgDOvwBHrxAJf2naGqbaiab=ri8fjabcUfaBnaarababaGaeiikaGIaemiDaqNaey4kaSIaeGymaeJaeiykaKIaeiyxa0faleaacqWGPbqAdaWgaaadbaGaeGymaedabeaaliabcYcaSiabdgfarjabcYcaSiabdMgaPnaaBaaameaacqaIYaGmaeqaaaWcbeqdcqGHpis1aaGcbaGaeyypa0dabaGaeiikaGIaeGymaeJaeyOeI0Iaem4yam2aaSbaaSqaaiabigdaXaqabaGccqGGPaqkcqGGOaakcqaIXaqmcqGHsislcqWGJbWydaWgaaWcbaGaeGOmaidabeaakiabcMcaPiab=ri8fjabcUfaBnaarababaGaeiikaGIaemiDaqNaeiykaKIaeiyxa0Laey4kaSccciGae4NSdi2aaSbaaSqaaiabikdaYaqabaGccqWGJbWydaWgaaWcbaGaeGymaedabeaakiabcIcaOiabigdaXiabgkHiTiabdogaJnaaBaaaleaacqaIYaGmaeqaaOGaeiykaKYaaWbaaSqabeaacqWG0baDcqGHRaWkcqaIXaqmaaGccqGHRaWkcqGFYoGydaWgaaWcbaGaeGymaedabeaakiabdogaJnaaBaaaleaacqaIYaGmaeqaaOGaeiikaGIaeGymaeJaeyOeI0Iaem4yam2aaSbaaSqaaiabigdaXaqabaGccqGGPaqkdaahaaWcbeqaaiabdsha0jabgUcaRiabigdaXaaaaeaacqWGPbqAdaWgaaadbaGaeGymaedabeaaliabcYcaSiabdgfarjabcYcaSiabdMgaPnaaBaaameaacqaIYaGmaeqaaaWcbeqdcqGHpis1aaGcbaaabaGaey4kaScabaGaeiikaGIaem4yam2aaSbaaSqaaiabigdaXaqabaGccqGGOaakcqaIXaqmcqGHsislcqWGJbWydaWgaaWcbaGaeGOmaidabeaakiabcMcaPiabgUcaRiabdogaJnaaBaaaleaacqaIYaGmaeqaaOGaeiikaGIaeGymaeJaeyOeI0Iaem4yam2aaSbaaSqaaiabigdaXaqabaGccqGGPaqkcqGHRaWkcqWGJbWydaWgaaWcbaGaeGymaedabeaakiabdogaJnaaBaaaleaacqaIYaGmaeqaaOGaeiykaKYaaebeaeaacqGGOaakcqaIWaamcqGGPaqkdaqeqaqaamaarababaaaleaacqWGPbqAdaWgaaadbaGaeGOmaidabeaaaSqab0Gaey4dIunaaSqaaiabdMgaPnaaBaaameaacqaIXaqmaeqaaaWcbeqdcqGHpis1aaWcbaGaemyuaefabeqdcqGHpis1aaaakiaaxMaacaWLjaWaaeWaaeaacqaI4aaoaiaawIcacaGLPaaaaaa@BD96@

This is a recurrence relationship that can be solved easily. We can prove that if (*u*_*t*_)_*t *≥ 0 _is a series in ℝ defined by

*u*_*t*+1 _= *au*_*t *_+ *bα*^*t*+1 ^+ *cγ*^*t*+1 ^+ *d*

then for every *t *≥ 0,

ut=atu0+b∑s=1tat−sαs+c∑s=1tat−sγs+d1−at1−a
 MathType@MTEF@5@5@+=feaafiart1ev1aaatCvAUfKttLearuWrP9MDH5MBPbIqV92AaeXatLxBI9gBaebbnrfifHhDYfgasaacH8akY=wiFfYdH8Gipec8Eeeu0xXdbba9frFj0=OqFfea0dXdd9vqai=hGuQ8kuc9pgc9s8qqaq=dirpe0xb9q8qiLsFr0=vr0=vr0dc8meaabaqaciaacaGaaeqabaqabeGadaaakeaacqWG1bqDdaWgaaWcbaGaemiDaqhabeaakiabg2da9iabdggaHnaaCaaaleqabaGaemiDaqhaaOGaemyDau3aaSbaaSqaaiabicdaWaqabaGccqGHRaWkcqWGIbGydaaeWbqaaiabdggaHnaaCaaaleqabaGaemiDaqNaeyOeI0Iaem4CamhaaGGacOGae8xSde2aaWbaaSqabeaacqWGZbWCaaaabaGaem4CamNaeyypa0JaeGymaedabaGaemiDaqhaniabggHiLdGccqGHRaWkcqWGJbWydaaeWbqaaiabdggaHnaaCaaaleqabaGaemiDaqNaeyOeI0Iaem4CamhaaOGae83SdC2aaWbaaSqabeaacqWGZbWCaaGccqGHRaWkcqWGKbazdaWcaaqaaiabigdaXiabgkHiTiabdggaHnaaCaaaleqabaGaemiDaqhaaaGcbaGaeGymaeJaeyOeI0IaemyyaegaaaWcbaGaem4CamNaeyypa0JaeGymaedabaGaemiDaqhaniabggHiLdaaaa@6448@

Applying this result with *a *= (1 - *c*_1_)(1 - *c*_2_), *b *= *β*_2_*c*_1_, *α *= 1 - *c*_2_, *c *= *β*_1_*c*_2_, *γ *= 1 - *c*_1_, and *d *= (*c*_1 _+ *c*_2 _- *c*_1_*c*_2_)∏_*Q*_(0) ∏i1
MathType@MTEF@5@5@+=feaafiart1ev1aaatCvAUfeBSjuyZL2yd9gzLbvyNv2Caerbhv2BYDwAHbqedmvETj2BSbqee0evGueE0jxyaibaiKI8=vI8tuQ8FMI8Gi=hEeeu0xXdbba9frFj0=OqFfea0dXdd9vqai=hGuQ8kuc9pgc9s8qqaq=dirpe0xb9q8qiLsFr0=vr0=vr0dc8meaabaqaciGacaGaaeqabaqadeqadaaakeaadaqeqaqaaaWcbaGaamyAamaaBaaameaacaaIXaaabeaaaSqab0Gaey4dIunaaaa@36E0@∏i2
MathType@MTEF@5@5@+=feaafiart1ev1aaatCvAUfeBSjuyZL2yd9gzLbvyNv2CaerbwvMCKfMBHbqedmvETj2BSbqee0evGueE0jxyaibaieYdOi=BH8vipeYdI8qiW7rqqrFfpeea0xe9Lq=Jc9vqaqpepm0xbbG8FasPYRqj0=yi0lXdbba9pGe9qqFf0dXdHuk9fr=xfr=xfrpiWZqaaeaabiGaaiaacaqabeaabeqacmaaaOqaamaarababaaaleaacaWGPbWaaSbaaWqaaiaaikdaaeqaaaWcbeqdcqGHpis1aaaa@3376@ yields

E[∏i1,Q,i2(t)]=(1−c1)t(1−c2)t∏i1,Q,i2(0)+β2c1(1−c2)t(∑s=1t(1−c1)t−s)+β1c2(1−c1)t(∑s=1t(1−c2)t−s)+(c1+c2−c1c2)∏Q(0)1−(1−c1)t(1−c2)t1−(1−c1)(1−c2)∏i1∏i2=(1−c1)t(1−c2)t∏i1,Q,i2(0)+β2(1−c2)t(1−(1−c1)t)+β1(1−c1)t(1−(1−c2)t)+∏Q(0)(1−(1−c1)t(1−c2)t)∏i1∏i2
MathType@MTEF@5@5@+=feaafiart1ev1aaatCvAUfKttLearuWrP9MDH5MBPbIqV92AaeXatLxBI9gBamrtHrhAL1wy0L2yHvtyaeHbnfgDOvwBHrxAJfwnaebbnrfifHhDYfgasaacH8akY=wiFfYdH8Gipec8Eeeu0xXdbba9frFj0=OqFfea0dXdd9vqai=hGuQ8kuc9pgc9s8qqaq=dirpe0xb9q8qiLsFr0=vr0=vr0dc8meaabaqaciaacaGaaeqabaWaaeGaeaaakeaafaqaaeGbdaaaaeaat0uy0HwzTfgDPnwy3aqeh0uy0HwzTfgDPnwy3aacfaGae8hHWxKaei4waS1aaebeaeaacqGGOaakcqWG0baDcqGGPaqkcqGGDbqxaSqaaiabdMgaPnaaBaaameaacqaIXaqmaeqaaSGaeiilaWIaemyuaeLaeiilaWIaemyAaK2aaSbaaWqaaiabikdaYaqabaaaleqaniabg+GivdaakeaacqGH9aqpaeaacqGGOaakcqaIXaqmcqGHsislcqWGJbWydaWgaaWcbaGaeGymaedabeaakiabcMcaPmaaCaaaleqabaGaemiDaqhaaOGaeiikaGIaeGymaeJaeyOeI0Iaem4yam2aaSbaaSqaaiabikdaYaqabaGccqGGPaqkdaahaaWcbeqaaiabdsha0baakmaarababaGaeiikaGIaeGimaaJaeiykaKcaleaacqWGPbqAdaWgaaadbaGaeGymaedabeaaliabcYcaSiabdgfarjabcYcaSiabdMgaPnaaBaaameaacqaIYaGmaeqaaaWcbeqdcqGHpis1aaGcbaaabaGaey4kaScabaacciGae4NSdi2aaSbaaSqaaiabikdaYaqabaGccqWGJbWydaWgaaWcbaGaeGymaedabeaakiabcIcaOiabigdaXiabgkHiTiabdogaJnaaBaaaleaacqaIYaGmaeqaaOGaeiykaKYaaWbaaSqabeaacqWG0baDaaGcdaqadaqaamaaqahabaGaeiikaGIaeGymaeJaeyOeI0Iaem4yam2aaSbaaSqaaiabigdaXaqabaGccqGGPaqkdaahaaWcbeqaaiabdsha0jabgkHiTiabdohaZbaaaeaacqWGZbWCcqGH9aqpcqaIXaqmaeaacqWG0baDa0GaeyyeIuoaaOGaayjkaiaawMcaaiabgUcaRiab+j7aInaaBaaaleaacqaIXaqmaeqaaOGaem4yam2aaSbaaSqaaiabikdaYaqabaGccqGGOaakcqaIXaqmcqGHsislcqWGJbWydaWgaaWcbaGaeGymaedabeaakiabcMcaPmaaCaaaleqabaGaemiDaqhaaOWaaeWaaeaadaaeWbqaaiabcIcaOiabigdaXiabgkHiTiabdogaJnaaBaaaleaacqaIYaGmaeqaaOGaeiykaKYaaWbaaSqabeaacqWG0baDcqGHsislcqWGZbWCaaaabaGaem4CamNaeyypa0JaeGymaedabaGaemiDaqhaniabggHiLdaakiaawIcacaGLPaaaaeaaaeaacqGHRaWkaeaacqGGOaakcqWGJbWydaWgaaWcbaGaeGymaedabeaakiabgUcaRiabdogaJnaaBaaaleaacqaIYaGmaeqaaOGaeyOeI0Iaem4yam2aaSbaaSqaaiabigdaXaqabaGccqWGJbWydaWgaaWcbaGaeGOmaidabeaakiabcMcaPmaarababaGaeiikaGIaeGimaaJaeiykaKcaleaacqWGrbquaeqaniabg+GivdGcdaWcaaqaaiabigdaXiabgkHiTiabcIcaOiabigdaXiabgkHiTiabdogaJnaaBaaaleaacqaIXaqmaeqaaOGaeiykaKYaaWbaaSqabeaacqWG0baDaaGccqGGOaakcqaIXaqmcqGHsislcqWGJbWydaWgaaWcbaGaeGOmaidabeaakiabcMcaPmaaCaaaleqabaGaemiDaqhaaaGcbaGaeGymaeJaeyOeI0IaeiikaGIaeGymaeJaeyOeI0Iaem4yam2aaSbaaSqaaiabigdaXaqabaGccqGGPaqkcqGGOaakcqaIXaqmcqGHsislcqWGJbWydaWgaaWcbaGaeGOmaidabeaakiabcMcaPaaadaqeqaqaamaarababaaaleaacqWGPbqAdaWgaaadbaGaeGOmaidabeaaaSqab0Gaey4dIunaaSqaaiabdMgaPnaaBaaameaacqaIXaqmaeqaaaWcbeqdcqGHpis1aaGcbaaabaGaeyypa0dabaGaeiikaGIaeGymaeJaeyOeI0Iaem4yam2aaSbaaSqaaiabigdaXaqabaGccqGGPaqkdaahaaWcbeqaaiabdsha0baakiabcIcaOiabigdaXiabgkHiTiabdogaJnaaBaaaleaacqaIYaGmaeqaaOGaeiykaKYaaWbaaSqabeaacqWG0baDaaGcdaqeqaqaaiabcIcaOiabicdaWiabcMcaPaWcbaGaemyAaK2aaSbaaWqaaiabigdaXaqabaWccqGGSaalcqWGrbqucqGGSaalcqWGPbqAdaWgaaadbaGaeGOmaidabeaaaSqab0Gaey4dIunaaOqaaaqaaiabgUcaRaqaaiab+j7aInaaBaaaleaacqaIYaGmaeqaaOGaeiikaGIaeGymaeJaeyOeI0Iaem4yam2aaSbaaSqaaiabikdaYaqabaGccqGGPaqkdaahaaWcbeqaaiabdsha0baakmaabmaabaGaeGymaeJaeyOeI0IaeiikaGIaeGymaeJaeyOeI0Iaem4yam2aaSbaaSqaaiabigdaXaqabaGccqGGPaqkdaahaaWcbeqaaiabdsha0baaaOGaayjkaiaawMcaaiabgUcaRiab+j7aInaaBaaaleaacqaIXaqmaeqaaOGaeiikaGIaeGymaeJaeyOeI0Iaem4yam2aaSbaaSqaaiabigdaXaqabaGccqGGPaqkdaahaaWcbeqaaiabdsha0baakmaabmaabaGaeGymaeJaeyOeI0IaeiikaGIaeGymaeJaeyOeI0Iaem4yam2aaSbaaSqaaiabikdaYaqabaGccqGGPaqkdaahaaWcbeqaaiabdsha0baaaOGaayjkaiaawMcaaaqaaaqaaiabgUcaRaqaamaarababaGaeiikaGIaeGimaaJaeiykaKcaleaacqWGrbquaeqaniabg+GivdGccqGGOaakcqaIXaqmcqGHsislcqGGOaakcqaIXaqmcqGHsislcqWGJbWydaWgaaWcbaGaeGymaedabeaakiabcMcaPmaaCaaaleqabaGaemiDaqhaaOGaeiikaGIaeGymaeJaeyOeI0Iaem4yam2aaSbaaSqaaiabikdaYaqabaGccqGGPaqkdaahaaWcbeqaaiabdsha0baakiabcMcaPmaarababaWaaebeaeaaaSqaaiabdMgaPnaaBaaameaacqaIYaGmaeqaaaWcbeqdcqGHpis1aaWcbaGaemyAaK2aaSbaaWqaaiabigdaXaqabaaaleqaniabg+Givdaaaaaa@56B5@

Replacing *β*_1 _and *β*_2 _by their actual expressions gives

E[∏i1,Q,i2(t)]=∏Q(0)∏i1∏i2+(1−c1)t(∏i1,Q(0)−∏Q(0)∏i1)∏i2+ (1− c2)t(∏Q,i2(0)−∏Q(0)∏i2)∏i1+(1−c1)t(1−c2)t(∏i1,Q,i2(0)−∏i1,Q(0)∏i2−∏Q,i2(0)∏i1+∏Q(0)∏i1∏i2)     (9)
MathType@MTEF@5@5@+=feaafiart1ev1aaatCvAUfKttLearuWrP9MDH5MBPbIqV92AaeXatLxBI9gBamrtHrhAL1wy0L2yHvtyaeHbnfgDOvwBHrxAJfwnaebbnrfifHhDYfgasaacH8akY=wiFfYdH8Gipec8Eeeu0xXdbba9frFj0=OqFfea0dXdd9vqai=hGuQ8kuc9pgc9s8qqaq=dirpe0xb9q8qiLsFr0=vr0=vr0dc8meaabaqaciaacaGaaeqabaWaaeGaeaaakeaafaqaaeGadaaabaWenfgDOvwBHrxAJf2naeXbnfgDOvwBHrxAJf2naGqbaiab=ri8fjabcUfaBnaarababaGaeiikaGIaemiDaqNaeiykaKIaeiyxa0faleaacqWGPbqAdaWgaaadbaGaeGymaedabeaaliabcYcaSiabdgfarjabcYcaSiabdMgaPnaaBaaameaacqaIYaGmaeqaaaWcbeqdcqGHpis1aaGcbaGaeyypa0dabaWaaebeaeaacqGGOaakcqaIWaamcqGGPaqkaSqaaiabdgfarbqab0Gaey4dIunakmaarababaWaaebeaeaacqGHRaWkcqGGOaakcqaIXaqmcqGHsislcqWGJbWydaWgaaWcbaGaeGymaedabeaakiabcMcaPmaaCaaaleqabaGaemiDaqhaaOGaeiikaGYaaebeaeaacqGGOaakcqaIWaamcqGGPaqkaSqaaiabdMgaPnaaBaaameaacqaIXaqmaeqaaSGaeiilaWIaemyuaefabeqdcqGHpis1aOGaeyOeI0YaaebeaeaacqGGOaakcqaIWaamcqGGPaqkaSqaaiabdgfarbqab0Gaey4dIunakmaarababaGaeiykaKYaaebeaeaacqGHRaWkaSqaaiabdMgaPnaaBaaameaacqaIYaGmaeqaaaWcbeqdcqGHpis1aOGaeeiiaaIaeiikaGIaeGymaeJaeyOeI0caleaacqWGPbqAdaWgaaadbaGaeGymaedabeaaaSqab0Gaey4dIunakiabbccaGiabdogaJnaaBaaaleaacqaIYaGmaeqaaOGaeiykaKYaaWbaaSqabeaacqWG0baDaaGccqGGOaakdaqeqaqaaiabcIcaOiabicdaWiabcMcaPiabgkHiTmaarababaGaeiikaGIaeGimaaJaeiykaKYaaebeaeaacqGGPaqkdaqeqaqaaaWcbaGaemyAaK2aaSbaaWqaaiabigdaXaqabaaaleqaniabg+GivdaaleaacqWGPbqAdaWgaaadbaGaeGOmaidabeaaaSqab0Gaey4dIunaaSqaaiabdgfarbqab0Gaey4dIunaaSqaaiabdgfarjabcYcaSiabdMgaPnaaBaaameaacqaIYaGmaeqaaaWcbeqdcqGHpis1aaWcbaGaemyAaK2aaSbaaWqaaiabikdaYaqabaaaleqaniabg+GivdaaleaacqWGPbqAdaWgaaadbaGaeGymaedabeaaaSqab0Gaey4dIunaaOqaaaqaaiabgUcaRaqaaiabcIcaOiabigdaXiabgkHiTiabdogaJnaaBaaaleaacqaIXaqmaeqaaOGaeiykaKYaaWbaaSqabeaacqWG0baDaaGccqGGOaakcqaIXaqmcqGHsislcqWGJbWydaWgaaWcbaGaeGOmaidabeaakiabcMcaPmaaCaaaleqabaGaemiDaqhaaOGaeiikaGYaaebeaeaacqGGOaakcqaIWaamcqGGPaqkaSqaaiabdMgaPnaaBaaameaacqaIXaqmaeqaaSGaeiilaWIaemyuaeLaeiilaWIaemyAaK2aaSbaaWqaaiabikdaYaqabaaaleqaniabg+GivdGccqGHsisldaqeqaqaaiabcIcaOiabicdaWiabcMcaPmaarababaGaeyOeI0YaaebeaeaacqGGOaakcqaIWaamcqGGPaqkaSqaaiabdgfarjabcYcaSiabdMgaPnaaBaaameaacqaIYaGmaeqaaaWcbeqdcqGHpis1aaWcbaGaemyAaK2aaSbaaWqaaiabikdaYaqabaaaleqaniabg+GivdGcdaqeqaqaaiabgUcaRaWcbaGaemyAaK2aaSbaaWqaaiabigdaXaqabaaaleqaniabg+GivdGcdaqeqaqaaiabcIcaOiabicdaWiabcMcaPaWcbaGaemyuaefabeqdcqGHpis1aOWaaebeaeaadaqeqaqaaiabcMcaPaWcbaGaemyAaK2aaSbaaWqaaiabikdaYaqabaaaleqaniabg+GivdaaleaacqWGPbqAdaWgaaadbaGaeGymaedabeaaaSqab0Gaey4dIunaaSqaaiabdMgaPjabigdaXiabcYcaSiabdgfarbqab0Gaey4dIunaaaGccaWLjaGaaCzcamaabmaabaGaeGyoaKdacaGLOaGaayzkaaaaaa@F559@

## Figures and Tables

**Table 1:** General Comparison between T2 and HAPim.

Marker type	*N*	Marker spacing	MSE		Difference in MSE P value
				
			T2	HAPim	
SNP^(1)^	200	2 cM	5.10	5.08	0.948
	400	2 cM	4.69	5.04	0.366
	200	0.25 cM	2.00	1.24	< 0.001**
	400	0.25 cM	1.34	0.92	0.005**

MST^(2)^	200	2 cM	2.93	2.77	0.438
	400	2 cM	1.81	1.44	0.056
	200	0.25 cM	0.71	0.46	0.012*
	400	0.25 cM	0.49	0.30	0.033*

* : P < 0.05, ** : P < 0.01

^(1) ^: single nucleotide polymorphism

^(2) ^: microsatellite

Mean square errors (MSEs) in cM^2 ^of quantitative trait locus (QTL) location estimates obtained by the T2 and HAPim methods for various effective population sizes, marker spacings and marker types, *t *= 100 and the initial association was complete.

**Table 2:** Comparison of model parameter estimates.

Model parameter	True value	Empirical mean (standard error)	
		
		T2	HAPim
*x *(in cM)	3.6	3.73 (5.1e-2)	3.62 (4.3e-2)
∏_*Q*_(0)	0.00125	0.13 (3.4e-3)	0.12 (3.0e-3)
*a*	1	1.02 (2.5e-2)	0.98 (2.4e-2)
*d*	1	0.87 (3.2e-2)	0.97 (2.7e-2)
*t*	100	57.9 (8.5)	53.4 (4.6)
α	1	0.92 (6.3e-3)	0.92 (6.0e-3)

Empirical means (and their standard errors) of the model parameter estimates under the T2 and HAPim methods. The single nucleotide polymorphism (SNP) marker spacing was 0.25 cM, the effective population size was *N *= 400, and the initial association was complete.

**Table 3:** Effect of effective population size.

*N*	MSE		Difference in MSE P value
		
	T2	HAPim	
200	0.63	0.44	0.001**
400	0.52	0.44	0.135
800	0.30	0.29	0.782
1600	0.15	0.13	0.414

**: P < 0.01

Mean square errors (MSEs) in cM^2 ^of quantitative trait locus (QTL) location estimates obtained by the T2 and HAPim methods for various effective population sizes. The single nucleotide polymorphism (SNP) marker spacing was 0.125 cM, *t *= 100 and the initial association was complete.

**Table 4:** Effect of sample size.

*N*	*N*_*s*_	MSE		Difference in MSE P value	Power	
				
		T2	HAPim		T2	HAPim
400	50	1.34	0.99	< 0.001**	0.36	0.59
	100	1.07	0.84	0.011*	0.66	0.88
	200	0.74	0.56	0.013*	0.88	0.99

800	100	1.08	0.87	0.033*	0.44	0.69
	200	0.66	0.49	0.008**	0.83	0.95
	400	0.42	0.31	0.006**	0.99	1.00

* : P < 0.05, ** : P < 0.01

Mean square errors (MSEs) in cM^2 ^of quantitative trait locus (QTL) location estimates and powers to detect the QTL obtained by the T2 and HAPim methods for various population and sample sizes. The single nucleotide polymorphism (SNP) marker spacing was 0.125 cM, *t *= 100, and the initial association was complete. The power was computed for a type I error of 0.05.

**Table 5:** Effect of time since initial creation of linkage disequilibrium (LD).

*t*	MSE		Difference in MSE P value
		
	T2	HAPim	
50	0.69	0.64	0.495
100	0.52	0.44	0.135
200	0.41	0.26	< 0.001**
300	0.25	0.17	0.005**

*: P < 0.05, ** : P < 0.01

Mean square errors (MSEs) in cM^2 ^of quantitative trait locus (QTL) location estimates obtained by the T2 and HAPim methods for various values of time *t *since initial LD creation. The single nucleotide polymorphism (SNP) marker spacing was 0.125 cM, *N *= 400, and the initial association was complete.

**Table 6:** Incomplete initial linkage disequilibrium (LD) scenario.

Marker type	MSE		Difference in MSE P value
		
	T2	HAPim	
Initial frequency of *Q *= 5%			

SNP^(1)^	0.99	0.68	0.031*
MST^(2)^	0.42	0.17	< 0.001**

Initial frequency of *Q *= 10%			

SNP^(1)^	0.95	0.64	0.039*
MST^(2)^	0.63	0.20	< 0.001**

* : P < 0.05, ** : P < 0.01

^(1) ^: single nucleotide polymorphism

^(2) ^: microsatellite

Mean square errors (MSEs) in cM^2 ^of quantitative trait locus (QTL) location estimates obtained by the T2 and HAPim methods for various heterogeneity parameter values, *t *= 100, *N *= 400, and a marker spacing of 0.25 cM.

**Table 7:** Scenario with phenocopies.

Marker type	MSE		Difference in MSE P value
		
	T2	HAPim	
Phenocopy rate = 15% ^*b*^			

SNP^(1)^	2.65	2.03	0.021*
MST^(2)^	0.84	0.35	< 0.001**

Phenocopy rate = 30%			

SNP^(1)^	4.90	3.29	< 0.001**
MST^(2)^	1.94	0.67	< 0.001**

* : P < 0.05, ** : P < 0.01

^*b*^: Phenocopy rate refers to the percentage of *q *alleles in the last generation that have given the same phenotype as the *Q *allele

^(1) ^: single nucleotide polymorphism

^(2) ^: microsatellite

Mean square errors (MSEs) in cM^2 ^of quantitative trait locus (QTL) location estimates obtained by the T2 and HAPim methods for various phenocopy rate values, *t *= 100, *N *= 400, and a marker spacing of 0.25 cM.

**Table 8:** Comparison with the IBD method of Meuwissen and Goddart.

Marker spacing (cM)	Deviation				
	
	0	1	2	3	4
frequency of allele Q ≥ 0.1					

1.0	16	17	9	5	3
0.5	12	20	10	2	6
0.25	12	18	8	6	6

frequency of allele Q ≥ 0					

1.0	15	14	6	8	7
0.5	10	17	12	7	4
0.25	11	14	11	5	9

Distribution of the deviations (in marker brackets) of the quantitative trait locus (QTL) location estimates from the correct bracket for the HAPim method under the default simulation scenarios (biallelic markers with *N *= 100, *N*_*s *_= 100, and *t *= 100) described in [1]. A deviation of 0 means the estimated position was in the correct marker bracket, 1 means the estimated position was one bracket away from the correct position, etc.
